# Systematic Evaluation of CLIP, LLM2CLIP, and LLaVA-Enhanced CLIP for Multimodal Disaster Classification

**DOI:** 10.3390/s26217047

**Published:** 2026-11-07

**Authors:** Hasibullah Mohmand, Zeynep Hilal Kilimci, Ayhan Küçükmanisa

**Affiliations:** 1 Department of Information Systems Engineering, Kocaeli University, Kocaeli 41001, Turkey; hasibullah.mohmand13@gmail.com (H.M.); zeynep.kilimci@kocaeli.edu.tr (Z.H.K.); 2 Department of Electronics and Communication Engineering, Kocaeli University, Kocaeli 41001, Turkey; 3 EVSTEK Information Technology Company, Kocaeli 41275, Turkey

**Keywords:** multimodal disaster classification, CrisisMMD, vision–language models, CLIP, LLM2CLIP, cross-modal fusion

## Abstract

Disaster-related social media posts combine images and text, but the benefits of large language model (LLM)-derived representations for crisis classification remain uncertain. We evaluate three pretrained vision–language pipelines—CLIP, LLM2CLIP, and LLaVA-enhanced CLIP—on CrisisMMD informativeness, humanitarian, and image-derived damage-severity classification. Development experiments examine fusion and class-imbalance strategies; frozen-feature probes assess representation pathways, and selected configurations undergo five-seed held-out testing. CLIP achieves test macro-F1 of 92.32±0.55% and 87.52±1.09% for informativeness and humanitarian classification, respectively; development-selected LLaVA-enhanced CLIP achieves 64.06±0.98% for damage severity. Exact seed-blocked tests show no significant differences among pipeline families after Holm correction across nine contrasts (minimum adjusted p=0.5625). Frozen-feature probes show no consistent predictive advantage for LLM2CLIP-derived text under matched visual-feature controls. Because the released damage-severity partitions share tweet identifiers across splits, we also conduct five-seed tweet-disjoint retraining, yielding macro-F1 between 62.46% and 63.80% across the pipelines. A matched frozen CLIP control shows negligible change from adding the original tweet to the image input. Image–text retrieval, exploratory cross-event evaluation, and computational profiling characterize task-dependent alignment, transfer, and resource trade-offs. The study provides a disaster-domain comparison of established pipelines without isolating the effects of LLM-derived representations or establishing universal model-family superiority.

## 1. Introduction

Natural disasters cause substantial human and socioeconomic losses worldwide, highlighting the importance of timely information collection and situational awareness during emergency response [1]. Rapid assessment of affected populations, infrastructure damage, and emerging humanitarian needs is particularly important during the initial stages of a disaster [2,3]. Social media platforms provide valuable sources of crisis-related information through user-generated text and images. However, these reports are often noisy, incomplete, ambiguous, or irrelevant, making the automated identification and classification of actionable information a challenging task [4,5].

Previous studies have investigated disaster-related social media classification using textual models such as BERT [6], visual architectures including VGG19 and ResNet [7], and multimodal approaches that combine textual and visual representations through different fusion strategies [8,9,10]. More recent studies have explored semi-supervised learning and task-specific multimodal architectures, including FixMatchLS [11] and Multihead APM [12]. These approaches demonstrate the potential of multimodal learning for crisis classification, although their supervision requirements, model architectures, and evaluation protocols differ.

Advances in pretrained vision–language models have introduced new opportunities for multimodal representation learning. CLIP [13] learns visual and textual representations within a shared embedding space through contrastive pretraining, enabling image–text similarity estimation and transfer to downstream tasks. Building upon this paradigm, LLM2CLIP [14] incorporates large language model (LLM)-derived text representations to enhance the linguistic capabilities of vision–language systems. Meanwhile, large multimodal models such as LLaVA [15] enable image-conditioned text generation, providing an alternative approach to incorporating visual information into textual representations. Despite these advances, more expressive language representations and increasingly complex multimodal processing do not necessarily guarantee better performance in domain-specific classification tasks.

In disaster-related social media analysis, the comparative effectiveness of these pretrained approaches requires further investigation. Specifically, we examine whether pipelines incorporating LLM-derived text features or image-conditioned text enrichment offer consistent advantages over a CLIP-based pipeline across disaster classification tasks. Because the systems differ in visual and textual components, the main comparisons assess complete pipelines rather than the isolated contribution of a text encoder. Image–text similarity and retrieval, transfer to selected unseen events, and computational cost provide complementary evidence beyond classification performance.

Motivated by these considerations, this study systematically evaluates three existing pretrained vision–language pipelines: CLIP, LLM2CLIP, and LLaVA-enhanced CLIP. The CLIP baseline directly encodes the original tweet and its corresponding image, whereas LLM2CLIP employs pretrained LLM-derived text representations and its associated visual encoder. The LLaVA-enhanced CLIP pipeline uses a frozen LLaVA model to generate image-conditioned textual descriptions before encoding the resulting text and original image with CLIP. The extracted representations are combined using established feature-level fusion mechanisms and passed to a multilayer perceptron classifier. We investigate selective encoder fine-tuning, class-imbalance handling, and three fusion strategies: concatenation, learnable weighted fusion, and feature-wise gated fusion.

The evaluation uses the CrisisMMD benchmark [16] for informativeness detection (Task 1), humanitarian categorization (Task 2), and damage-severity assessment (Task 3). Tasks 1 and 2 follow the released agreed-label multimodal partitions. Task 3 uses image-derived severity labels with accompanying tweets as contextual input. To examine the predictive contribution of this context, we evaluate separate frozen-encoder modality controls, including a matched frozen CLIP image-only versus image-plus-tweet comparison; these controls do not reproduce the final partially fine-tuned configurations. Model configurations are selected using development data and evaluated on held-out test sets across five training seeds. Further analyses assess representation ablations, image–text similarity and retrieval, transfer to three held-out events, tweet-disjoint Task 3 retraining, and computational cost.

The main contributions of this study are summarized as follows:

- We present a systematic evaluation of three pretrained vision–language pipelines—CLIP, LLM2CLIP, and LLaVA-enhanced CLIP—across the informativeness, humanitarian, and damage-severity classification tasks of CrisisMMD, examining their effectiveness under consistent within-study evaluation protocols.
- We investigate selective encoder fine-tuning, class-imbalance handling, and established feature-level fusion strategies. Additionally, a controlled frozen-feature ablation compares CLIP and LLM2CLIP text representations using a common visual backbone and matched downstream classifier capacity within each probe, providing evidence about their relative predictive contributions.
- We audit cross-partition tweet overlap in the released damage-severity benchmark and perform five-seed tweet-group-disjoint retraining on an unchanged test partition, assessing the sensitivity of classification results to this alternative protocol without claiming an incremental benefit from tweet text.
- We evaluate the selected model configurations across five training seeds, reporting held-out classification performance with variability estimates. Seed-blocked exact paired sign-flip tests, paired-seed bootstrap intervals, and Holm correction over all nine within-task comparisons provide exploratory uncertainty estimates. These analyses do not establish consistent model-family superiority.
- We examine cross-modal representation behavior through matched and mismatched image–text similarity measurements and image-to-text retrieval metrics, including Recall@1, Recall@5, and Recall@10. We further investigate the effect of LLaVA-based image-conditioned text enrichment on these measurements.
- We assess practical limitations through exploratory transfer evaluations on three selected held-out events and hardware-matched computational profiling, examining event-specific performance alongside model complexity, inference latency, GPU memory requirements, and throughput.

The remainder of this paper is organized as follows. Section 2 reviews related work on multimodal crisis classification. Section 3 describes the evaluated pretrained architectures, dataset, preprocessing procedures, and fusion mechanisms. Section 4 presents the experimental settings, evaluation protocols, and results. Section 5 discusses the main findings and limitations, while Section 6 concludes the study and outlines directions for future research.

## 2. Related Work

### 2.1. Unimodal Crisis-Content Classification

Automated classification of disaster-related social media content has been investigated using both textual and visual representations. The CrisisMMD dataset, introduced by Alam et al. [16], provides a multimodal benchmark for identifying informative content, categorizing humanitarian needs, and assessing disaster-related damage. Early approaches commonly considered textual and visual modalities independently, establishing reference methods for subsequent multimodal classification research.

Text-based approaches have employed conventional machine learning algorithms, feature-engineering techniques, and pretrained language models. The study in [6] compared multiple approaches for humanitarian text classification, including TF-IDF, Word2Vec, conventional classifiers, and fine-tuned BERT. The reported BERT model achieved 77.13% accuracy and 76.86% F1-score under a 70:30 train–test partition with SMOTE-based class balancing.

Other approaches have combined pretrained text embeddings with linguistic and metadata features. For example, Jain et al. [17] investigated text classification using pretrained embeddings together with sentiment, lexical, and metadata information, reporting 87.93% accuracy on an informativeness classification task.

Visual classification has similarly been explored using convolutional neural networks. The study in [7] evaluated VGG19, ResNet, InceptionV3, MobileNetV2, and XceptionNet for disaster-image informativeness classification. XceptionNet achieved a reported accuracy of 80.67% and F1-score of 80.64% under an 80:20 train–test partition.

These studies demonstrate the applicability of both conventional and deep learning approaches to crisis-related text and image classification. However, their unimodal input settings and evaluation protocols differ from those of the multimodal systems considered in this study. Consequently, their reported results provide methodological context rather than direct evidence of comparative performance.

### 2.2. Multimodal Fusion for Disaster Classification

Multimodal approaches combine textual and visual information to exploit complementary signals present in social media reports. The CrisisMMD multimodal baselines reported by Ofli et al. [18] combined a CNN-based text encoder with VGG16 visual representations and evaluated text-only, image-only, and multimodal configurations. Their reported multimodal F1-scores were 84.2% for informativeness detection and 78.3% for humanitarian classification. These results provide an important historical reference for subsequent research using the released benchmark.

Later studies investigated combinations of pretrained language models and visual feature extractors. Kota et al. [19] combined BERT text embeddings with RegNetY320 visual features and reported accuracies of 89.63% and 86.59% for informativeness and humanitarian classification, respectively.

Adwaith et al. [20] investigated multimodal disaster classification using combinations of pretrained textual encoders, including BERT, XLNet, and RoBERTa, and visual architectures such as ResNet, ResNeXt, and DenseNet. Their study addressed informativeness and humanitarian classification, although model-specific attribution of the available numerical results is insufficient for inclusion in the benchmark comparison. Similarly, Kamoji et al. [9] combined BERT and DenseNet-121 features for informativeness classification, reporting 85.33% accuracy and 85.09% F1-score.

Other studies examined alternative feature extraction and classification mechanisms. The approach in [21] combined VGG16 visual representations with FastText embeddings and evaluated several disaster-classification objectives. Its humanitarian analysis employed a custom two-stage protocol, while damage-severity results were evaluated using study-specific event-stratified partitions rather than the released fixed split used here.

The study in [22] investigated a hybrid architecture combining Vision Transformer representations, GPT-2-derived text features, and a random forest classifier. These approaches illustrate the variety of encoder combinations investigated for multimodal crisis analysis.

Beyond encoder selection, feature integration has been an important research direction. Tasneem et al. [8] investigated an early-fusion architecture combining BERT-LSTM textual representations with ResNet50 visual features. Their study reported higher performance for the evaluated early-fusion configuration than for its late-fusion comparator.

An earlier crisis-specific approach by Abavisani et al. [23] combined cross-attention, which selects useful information from heterogeneous image and text inputs, with stochastic multimodal graph-based training to improve robustness to incomplete and noisy paired information. Their reported SSE-Cross-BERT-DenseNet system was evaluated under multiple study-defined data settings, including a consistent-label setting that differs from the released partitions in our evaluation. The attention mechanism and graph-based training differ fundamentally from the feature-level fusion operators tested here.

Transformer-based multimodal integration has also been explored. Yoon et al. [24] introduced RoBERTaMFT, a multimodal fusion approach incorporating cross-modal information and co-learning for disaster classification. Such methods extend beyond conventional feature concatenation by learning additional interactions between modality representations.

Class imbalance and limited annotated data have motivated alternative training strategies. FixMatchLS [11] investigated semi-supervised multimodal learning, while Sîrbu et al. [12] proposed a multihead co-training approach incorporating pseudo-labeling and adaptive confidence mechanisms.

These studies demonstrate that multimodal disaster classification can benefit from different encoder, fusion, and learning strategies. Nevertheless, their supervision regimes, data partitions, label definitions, and reported metrics are not always equivalent. Performance comparisons across publications must therefore distinguish directly matched evaluations from contextual literature results.

### 2.3. Pretrained Vision–Language Models and Image-Conditioned Text Generation

Pretrained vision–language models provide an alternative to independently trained textual and visual encoders. CLIP, introduced by Radford et al. [13], uses contrastive image–text pretraining to learn representations in a shared embedding space. Its pretrained encoders support image–text similarity estimation and transfer to downstream visual and multimodal tasks.

Dinani and Caragea [25] investigated disaster-image classification using pretrained ViT, CSWin, ConvNeXt, and the visual encoder of CLIP (ViT-B/32). They evaluated supervised, zero-shot, few-shot, and transfer settings and reported average supervised weighted-F1 scores of 86.57% and 81.81% for informativeness and humanitarian classification across seven disaster events, respectively. Their evaluation used image-only inputs, event-specific partitions, and a different humanitarian label taxonomy; these scores therefore provide contextual evidence for pretrained image representations, not a directly comparable paired image–text macro-F1 benchmark.

A closely related CLIP-based method is CLIP-BCA-Gated, introduced by Li et al. [26] for CrisisMMD humanitarian categorization. It augments pretrained CLIP features with bidirectional cross-attention and adaptive modality gating. The authors reported 91.77% accuracy and 91.74% F1 (as reported, without assuming a macro-averaging definition), and presented attention and gating ablations. Their study describes stratified 70/15/15 splitting instead of the released fixed train/dev/test partitions used here. The resulting scores therefore provide methodological context, not a matched comparison with our Task 2 test macro-F1. Notably, the bidirectional cross-attention architecture differs from our global weighted fusion and feature-wise gating of pooled embeddings.

More recently, LLM2CLIP was introduced by Huang et al. [14] to incorporate large language model (LLM)-derived text representations into a vision–language alignment framework. Its pretrained architecture provides an approach to representing linguistic content using a substantially larger language backbone than conventional CLIP text encoders. However, improvements demonstrated on general vision–language benchmarks do not necessarily translate into better performance on specialized disaster-classification tasks. Furthermore, differences between pretrained model families may arise from both textual and visual representations, highlighting the importance of controlled component-level evaluation when investigating the contribution of LLM-derived text features.

Another research direction employs generative vision–language models to enrich the textual information available for classification. LLaVA, introduced by Liu et al. [15], supports image-conditioned language generation through multimodal instruction tuning.

Related disaster-classification studies have investigated caption-based and visually enriched text representations. Farah et al. [27] introduced Caption-based Multimodal BERT (CMB), which represents images through generated captions combined with tweet text. Their CMB-CLIP-cc variant reported 92.03% accuracy and 90.81% macro-F1 for informativeness, and 88.27% accuracy and 78.10% macro-F1 for humanitarian classification. These values are specific to that captioning variant and are distinct from the paper’s weighted-F1 scores.

Similarly, Giaccaglia et al. [28] investigated LLaVA-generated image descriptions combined with the original tweet and subsequently classified using RoBERTa. The reported LLaVA + RoBERTa configuration achieved 91.4% accuracy and 90.2% macro-F1 on the informativeness classification task.

These approaches indicate that pretrained vision–language representations and image-conditioned text generation are relevant alternatives for disaster-related classification. However, differences in model architecture, input construction, training protocols, and evaluation metrics complicate direct comparisons between published results.

### 2.4. Research Gap and Positioning

Previous research has established the effectiveness of unimodal learning, multimodal fusion, pretrained vision–language representations, and image-conditioned text generation for various crisis-classification tasks. However, these approaches differ in their model architectures, input representations, supervision requirements, and evaluation protocols, complicating direct comparisons of their reported performance.

In particular, the studies reviewed here do not establish whether LLM-derived textual representations consistently improve disaster classification relative to conventional CLIP representations, or whether image-conditioned text enrichment provides benefits across different crisis-classification objectives. Moreover, predictive performance alone cannot establish improvements in image–text semantic alignment. These considerations motivate evaluations that complement classification metrics with component-level comparisons and direct embedding-space measurements.

Building on these research directions, the present study evaluates CLIP, LLM2CLIP, and LLaVA-enhanced CLIP across three CrisisMMD classification tasks. The investigation combines supervised adaptation, established feature-level fusion strategies, controlled frozen-feature ablations, multi-seed statistical evaluation, image–text similarity and retrieval analysis, exploratory cross-event generalization, and computational profiling.

The contribution lies in the systematic evaluation of these existing approaches within the investigated disaster-domain setting rather than the introduction of a new pretrained architecture or fusion mechanism.

Because published CrisisMMD studies differ in their data preparation, supervision regimes, model configurations, and metric definitions, their reported results cannot automatically be interpreted as directly comparable. We therefore distinguish contextual literature comparisons from evaluations conducted under verified, equivalent experimental conditions.

## 3. Adaptation of Pretrained Vision–Language Models

We investigate three pretrained vision–language pipelines for supervised classification of paired crisis images and tweets: CLIP, LLM2CLIP, and LLaVA-enhanced CLIP.

The CLIP baseline uses openai/clip-vit-large-patch14-336 for visual and textual encoding.

LLM2CLIP combines the pretrained microsoft/LLM2CLIP-Llama-3-8B-Instruct-CC-Finetuned LLM2Vec-based text encoder and text adapter with the microsoft/LLM2CLIP-Openai-L-14-336 visual checkpoint.

LLaVA-enhanced CLIP first applies a frozen llava-hf/llava-1.5-7b-hf model to generate an image-conditioned rewrite of the source tweet; a separate pretrained CLIP model then encodes the original image and the rewritten text. In all three pipelines, the modality representations are independently L2-normalized, fused at the feature level, and classified by a two-layer multilayer perceptron (MLP). We evaluate established supervised adaptation strategies, including selective encoder fine-tuning and class-imbalance handling, without claiming a new pretrained architecture, alignment objective, or fusion operator. Hereafter, CLIP refers to the supervised OpenAI CLIP baseline, LLM2CLIP to the existing Microsoft pretrained model, and LLaVA-enhanced CLIP (LLaVA-CLIP in compact tables) to the text-rewriting pipeline followed by a separate CLIP classifier.

### 3.1. Dataset and Annotation Protocol

We use CrisisMMD [16], a multimodal dataset of disaster-related Twitter posts collected during seven natural disasters in 2017, including earthquakes, hurricanes, floods, and wildfires. The released collection contains approximately 18,082 images and 16,058 tweets with annotations supporting disaster-information analysis. Because tweets may contain multiple images, these corpus totals differ from the numbers of paired records available for each supervised task.

We consider three CrisisMMD classification tasks:

1. **Task 1: Informativeness (binary).** Paired posts are categorized as informative or not informative. The task identifies content potentially relevant to disaster response.
2. **Task 2: Humanitarian categorization.** The original eight annotation categories are consolidated into five categories in the released agreed-label multimodal benchmark:
  - Affected individuals;
  - Infrastructure and utility damage;
  - Rescue, volunteering, or donation effort;
  - Other relevant information;
  - Not humanitarian.
  The mapping from the original annotation categories is given in Table 1.
3. **Task 3: Damage severity (three classes).** Image-level annotations identify the following severity categories:
  - Severe damage;
  - Mild damage;
  - Little or no damage.
  The classification target is the image-derived severity label. The corresponding tweet is used as contextual input but has no independently annotated severity label.

The unit of observation in our supervised multimodal experiments is an image–tweet pair represented by a dataset row containing the image and its associated text. We preserve the source image_id and tweet_id identifiers. Since a tweet may be associated with more than one image, counts of paired records are not interchangeable with counts of unique tweets or separately annotated images. In Tasks 1 and 2, text and image annotations are assigned independently; Task 3 provides image-level severity annotations only.

For Tasks 1 and 2, we use the officially released CrisisMMD agreed-label multimodal training, development, and test TSV files. In both tasks, the independently assigned label_text and label_image annotations agree for each retained image–tweet pair. Task 1 addresses binary informativeness classification, distinguishing informative from not informative content. The released partitions contain 9601 training, 1573 development, and 1534 test image–tweet pairs, totaling 12,708 pairs across two classes. Task 2 addresses humanitarian categorization using the established five-class taxonomy provided in the agreed-label multimodal benchmark. Its partitions contain 6126 training, 998 development, and 955 test pairs, totaling 8079 pairs. The five humanitarian categories originate from the consolidation of eight original annotation categories performed during preparation of the released benchmark. Table 1 documents this original-to-five-class mapping, which was not introduced or modified by our study. For both tasks, our dataset loaders use the provided labels and fixed partitions without additional category merging, relabeling, row filtering, or random repartitioning during the final training runs.

The original CrisisMMD damage-severity annotations apply to images rather than their accompanying tweets [16]. Accordingly, Task 3 uses image-derived severity as the classification target and tweet text as contextual input; the dataset does not provide independent tweet-level severity labels with which to verify this correspondence. We use the released image-labeled TSV partitions, containing 2468 training, 529 development, and 529 test image–tweet pairs, for a total of 3526 records across the little/no, mild, and severe damage classes. The primary benchmark retains these image–tweet associations and partitions without relabeling or synthesizing additional severity annotations. Table 2 reports the class distributions for all three tasks; the separately trained Task 3 modality controls and tweet-disjoint sensitivity protocol are described in Section 4.4 and Section 4.6, respectively.

Paired records and distinct tweets.

Because a single tweet may contain multiple images, the number of image–tweet pairs can exceed the number of distinct tweets. The training, development, and test partitions contain 8293/1573/1534 unique tweets for Task 1; 5263/998/955 for Task 2; and 2286/519/523 for Task 3, respectively. Each image–tweet pair corresponds to a distinct image identifier within its partition. Consequently, the pair counts reported in Table 2 are not interchangeable with the numbers of unique tweets.

Pair integrity in Task 3.

All 3526 Task 3 records contain nonempty tweet text and image paths, and their image identifiers are unique. Each image path contains the stated image ID. For 3525 records, the image ID also begins with its associated tweet ID followed by the expected image index. One training record has a different tweet-ID prefix, although its image path and image ID are consistent. The semantic correspondence of this exceptional image–tweet pair has not been independently established. The released pairings were retained unchanged to preserve the benchmark protocol; identifier-level consistency should not be interpreted as independent confirmation of semantic alignment for every pair.

Independence of dataset partitions.

We compared tweet and image identifiers across the official partitions. Tasks 1 and 2 are both tweet disjoint and image disjoint across training, development, and test sets. Task 3 is image disjoint but not tweet disjoint: 62 distinct tweet IDs occur in both training and development, 62 in training and test, and 16 in development and test; 10 tweet IDs occur in all three partitions. These repeated IDs have identical source tweet text but correspond to different images. Consequently, 71 Task 3 development pairs and 67 test pairs share a tweet ID with a training pair. Repeated textual input creates a potential source of cross-partition information reuse in multimodal evaluation, even when every image identifier is unique. This is an identifier-overlap finding, not evidence that the paired tweets are semantically inconsistent with their images. These Task 3 partitions were released by the CrisisMMD dataset authors [16,18]; we retain them for benchmark comparability but do not characterize their fixed-split results as tweet-disjoint generalization. The observed identifier structure is consistent with image-level partition separation without tweet-level grouping; the original release does not document a tweet-group-disjoint splitting procedure, and this observation does not establish the authors’ exact splitting algorithm. To address this issue experimentally, we also report an independently retrained tweet-group-disjoint sensitivity protocol in Section 4.6; the original 529 test image–tweet pairs remain unchanged. The supplementary event-held-out experiment addresses a distinct transfer question and is not substituted for this leakage sensitivity study.

### 3.2. Data Preprocessing

Text preprocessing.

We apply the same deterministic normalization to source tweets across the three model families. Common malformed Unicode sequences are conservatively repaired only when the correction reduces suspicious encoding artifacts; an initial retweet marker (RT), HTTP/HTTPS URLs, and the # symbol are removed, while hashtag terms are retained and repeated whitespace is collapsed. Tokenization then follows the corresponding pretrained model: CLIP and the downstream LLaVA-enhanced CLIP classifier use their matching CLIP tokenizer with a maximum of 77 tokens; LLM2CLIP uses its LLM2Vec-compatible tokenizer, mean pooling, and a maximum of 512 tokens. The frozen LLaVA generator receives the normalized tweet together with the original image and produces image-conditioned text for subsequent CLIP tokenization.

Image preprocessing and visual checkpoints.

All final classification encoders process RGB images at 336×336 resolution using the appropriate CLIP image processor. CLIP and LLaVA-enhanced CLIP use the openai/clip-vit-large-patch14-336 checkpoint, while LLM2CLIP uses microsoft/LLM2CLIP-Openai-L-14-336 with the corresponding OpenAI CLIP image processor.

Although the final CLIP and LLM2CLIP pipelines both use ViT-L/14 visual encoders with 336×336 inputs, their pretrained visual weights differ. The main partially fine-tuned results therefore compare complete pipelines. In the separate frozen-feature analysis, identical OpenAI CLIP visual features and matched downstream classifier capacity are used to compare the predictive utility of the two text pathways (Section 4.4); differences in text architecture, tokenization, pretraining, and adapters remain.

For LLaVA-enhanced CLIP, the additional frozen generator processes the original RGB image with its own AutoProcessor; this generation-stage input processing is distinct from the 336×336 image input to the downstream CLIP classifier.

### 3.3. Evaluated Model Configurations

We evaluate three established pretrained representation families: CLIP [13], LLM2CLIP [14], and LLaVA-enhanced CLIP, which employs an image-conditioned text generator [15]. Each is adapted to supervised classification with the feature-level mechanisms described below. Relative to CLIP, LLM2CLIP differs in its text pathway, pretrained visual weights, and selected adaptation settings, while LLaVA-enhanced CLIP adds image-conditioned text generation before a separate CLIP classifier. Accordingly, differences in the principal benchmark results cannot be attributed exclusively to an individual text encoder.

Exploratory image-resolution comparison.

We evaluate 224×224 and 336×336 image-resolution variants on the development partitions using seed 42 for each model–task combination. At 224 pixels, CLIP and the downstream LLaVA-enhanced CLIP classifier use openai/clip-vit-large-patch14, and LLM2CLIP uses microsoft/LLM2CLIP-Openai-L-14-224. The final 336-pixel checkpoints are specified in the experimental settings. Because the resolution variants also differ in pretrained checkpoints, the experiment does not isolate the effect of image size. Only the 336-pixel configurations contribute to the five-seed benchmark, computational-profiling, cross-event, and fusion results; their use as a common final setting does not imply superior development performance for every model–task combination.

#### 3.3.1. CLIP (Contrastive Language–Image Pretraining)

CLIP [13] consists of visual and textual encoders trained contrastively on approximately 400 million image–text pairs. The visual tower may use a Vision Transformer or ResNet architecture, while the text tower uses a Transformer encoder. Both representations are projected into a shared embedding space and optimized so that paired examples have higher similarity than mismatched examples. This pretraining supports image–text similarity estimation, retrieval, and transfer to downstream tasks.

In our supervised setting, CLIP serves as the reference pipeline for evaluating the task-specific performance of the alternative systems. Its smaller text encoder and different pretraining objective do not, by themselves, establish a downstream disadvantage relative to LLM-based alternatives.

CLIP implementation and input pathway.

The CLIP baseline loads openai/clip-vit-large-patch14-336 through Hugging Face CLIPModel. Each normalized tweet is encoded with the matching CLIPTokenizerFast (maximum length 77; padding and truncation enabled), and the corresponding RGB image is processed using CLIPImageProcessor at 336×336. Features from the two pretrained towers are separately L2-normalized, concatenated, and classified by a 512-unit linear layer, ReLU activation, dropout (0.3), and a task-specific linear output layer. In the selected final configuration, the last five Transformer blocks of both CLIP towers and the classifier are trainable; the remaining encoder parameters are frozen. This supervised baseline uses the original paired tweet and image without label-description prompts or generated captions.

#### 3.3.2. LLM2CLIP

LLM2CLIP [14] is an existing vision–language approach that integrates representations from a pretrained large language model into a CLIP-compatible alignment framework. Its LLM-derived text pathway and pretrained visual model provide an alternative to the conventional CLIP text and visual towers. We adopt the publicly released Microsoft components rather than introducing a new LLM, adapter, or contrastive-pretraining objective.

The original LLM2CLIP study reports improvements on its own cross-modal retrieval benchmarks [14]. Such findings motivate evaluation in disaster informatics but do not establish corresponding advantages for CrisisMMD classification or intrinsic alignment. Our assessment therefore considers the model’s task-specific classification performance, image–text retrieval behavior, and computational requirements, without assuming that improvements observed in the original pretraining domain transfer to crisis-related tweets.

LLM2CLIP implementation and input pathway.

We load microsoft/LLM2CLIP-Llama-3-8B-Instruct-CC-Finetuned and its tokenizer into LLM2Vec, using pooling_mode = mean with a 512-token limit. The model encodes normalized source tweets without performing task-specific language generation. During partial fine-tuning, the implementation applies LLM2Vec’s prepare_for_tokenization and tokenize methods and executes a differentiable forward pass rather than the inference-only encode method, preserving gradients through the selected text layers. The pooled text representation is cast to the pretrained adapter’s parameter dtype before projection by the frozen Microsoft text adapter. In parallel, the image is encoded at 336×336 using microsoft/LLM2CLIP-Openai-L-14-336 with the matching CLIP image processor. Image and text features are independently L2-normalized, concatenated, and passed through the same MLP classifier design used for CLIP. The selected configuration fine-tunes the final three Transformer blocks of both the LLM2Vec text backbone and the visual backbone, along with the classifier; earlier blocks and the pretrained adapter remain frozen. The original LLM2CLIP contrastive pretraining is not repeated.

#### 3.3.3. LLaVA-Enhanced CLIP

The LLaVA-enhanced CLIP pipeline uses a frozen LLaVA generator to rewrite the original tweet using the paired image, followed by a separate supervised CLIP classifier. The generator is llava-hf/llava-1.5-7b-hf [15], loaded with AutoProcessor and LlavaForConditionalGeneration. It receives both the original image and normalized tweet; the resulting text is subsequently encoded with the CLIP text tower, while the original image is encoded independently with the visual tower of openai/clip-vit-large-patch14-336. LLaVA is used only for text enrichment and is neither the classification head nor a fine-tuned component of the downstream system.

The LLaVA model and processor were loaded from the Hugging Face repository using the default revision, without explicitly pinning a specific commit. For reference, the repository revision, https://huggingface.co/llava-hf/llava-1.5-7b-hf/commit/b234b804b114d9e37bb655e11cbbb5f5e971b7a9, accessed on 6 June 2025. However, since the exact revision used during the original experiments was not recorded, it cannot be retrospectively verified.

Rewriting prompt and multimodal input.

For image-conditioned text generation, the original image and normalized tweet are combined in a user-role message using the model processor’s chat template (add_generation_prompt=True). The prompt instruction is:

Using only information that is visible in the image or explicitly stated in the tweet, rewrite the tweet as one concise, factual description useful for crisis classification. Do not force a disaster interpretation when it is not supported. Do not invent people, locations, causes, damage, casualties, or other details that are not supported. Preserve uncertainty when the source is uncertain. Return only the rewritten text.

Original tweet: {tweet}

The placeholder {tweet} is replaced by the normalized source tweet. The paired image is loaded from its original path and converted to RGB. The processor receives both image and text inputs, so the generated reformulation is image-conditioned rather than a text-only paraphrase. If the processor does not support a chat template, the implementation uses an explicit <image>/USER:/ASSISTANT: prompt format.

Decoding, generated-text processing, and training.

Generation is deterministic, with do_sample=False and num_beams=1. We set max_new_tokens=64 and use_cache=True, processing one image–tweet pair per batch. Temperature, top-*p*, and repetition penalty are not explicitly set and therefore follow the checkpoint and software defaults where applicable. Prompt tokens are removed from the output; only newly generated tokens are decoded using skip_special_tokens=True and clean_up_tokenization_spaces=False. After trimming and collapsing whitespace, empty generations are replaced with the normalized source tweet. The output is tokenized by the CLIP tokenizer, with padding and truncation to a maximum of 77 tokens. The original image and enriched text are encoded by CLIP, separately L2-normalized, concatenated, and passed to the 512-unit ReLU–dropout (0.3)–linear classifier. In the selected final configuration, the last seven Transformer blocks of each downstream CLIP tower are trainable, while LLaVA remains frozen. Table 3 summarizes the generation and downstream classification settings.

Generated rewrites are stored in enriched_text_v2.json to avoid repeated generation for identical source pairs. Cache keys are SHA-256 hashes of the checkpoint name, prompt identifier, maximum generation length, image path, and normalized source tweet. The generator can be unloaded during supervised CLIP-classifier optimization and reloaded when required. Computational profiling disables generation-cache reuse to include the overhead of uncached image-conditioned rewriting.

### 3.4. Feature-Level Fusion and Classification

For each pretrained pipeline, let $v\in R^{d}$ and $t\in R^{d}$ denote its independently L2-normalized image and text embeddings. We compare three established feature-level fusion mechanisms:

(1) $$\begin{array}{cc} z_{\mathrm{concat}} & =[v;t], \end{array}$$

(2) $$\begin{array}{cc} \left(\alpha ,\beta \right) & =\mathrm{softmax}(a,b), \end{array}$$

(3) $$\begin{array}{cc} z_{\mathrm{weighted}} & =\alpha v+\beta t, \end{array}$$

(4) $$\begin{array}{cc} g & =\sigma (W\left[v;t\right]+b_{g}), \end{array}$$

(5) $$\begin{array}{cc} z_{\mathrm{gated}} & =g⊙v+(1-g)⊙t. \end{array}$$

Here, $z_{\mathrm{concat}}$, $z_{\mathrm{weighted}}$, and $z_{\mathrm{gated}}$ are fused feature vectors; *a* and *b* are scalar logits mapped by softmax to scalar modality weights α and β; and the weight matrix W and bias vector $b_{g}$ parameterize the sigmoid gate vector g.

Concatenation preserves both embedding vectors, whereas weighted fusion learns two global modality coefficients shared across samples and feature coordinates. Gated fusion computes an input-dependent coefficient for each feature dimension. The fused representation is classified using a 512-unit fully connected layer, ReLU activation, dropout (0.3), and a task-specific linear output layer. Concatenation produces a 2d-dimensional classifier input; weighted and gated fusion produce *d*-dimensional inputs, so classifier parameter counts are not identical across fusion mechanisms. These are established feature-level operations, not novel fusion architectures. In contrast to cross-attention-based approaches such as [23,26], the weighted and gated mechanisms operate on pooled embeddings and do not compute token- or patch-level bidirectional cross-attention. The single-seed development-set comparison in Section 4.2 supports the choice of concatenation for subsequent multi-seed evaluation but does not establish its universal superiority.

## 4. Experimental Results

We evaluate the three CrisisMMD classification tasks defined in Section 3.1 using accuracy, macro-averaged precision, recall, and F1, together with class-sensitive measures for imbalanced outcomes. Macro averaging assigns equal importance to each class, irrespective of its frequency.

For class *c*, precision, recall, and F1 are computed in a one-vs-rest formulation. Macro-F1 and balanced accuracy are defined as follows:

(6) $$\begin{array}{cc} P_{c} & =\frac{TP_{c}}{TP_{c}+FP_{c}},\;R_{c}=\frac{TP_{c}}{TP_{c}+FN_{c}}, \end{array}$$

(7) $$\begin{array}{cc} F1_{c} & =\frac{2P_{c}R_{c}}{P_{c}+R_{c}}, \end{array}$$

(8) $$\begin{array}{cc} F1_{\mathrm{macro}} & =\frac{1}{C}\sum_{c=1}^{C}F1_{c}, \end{array}$$

(9) $$\begin{array}{cc} \mathrm{BalancedAccuracy} & =\frac{1}{C}\sum_{c=1}^{C}R_{c}. \end{array}$$

Here *C* denotes the number of classes; undefined class-specific ratios are assigned zero. Balanced accuracy is therefore identical to macro recall in this multiclass setting. We also report uniformly averaged one-vs-rest ROC-AUC and macro AUPRC, the latter computed as the unweighted mean of one-vs-rest average-precision (AP) scores from predicted probabilities (sklearn.metrics.average_precision_score), rather than trapezoidal precision–recall area. Per-class metrics and confusion matrices provide additional information for imbalanced categories. Published results are presented separately from the controlled within-study comparisons in Section 4.11.

### 4.1. Experimental Settings

The three model families use the encoders listed in Table 4. CLIP and LLM2CLIP encode the cleaned tweet directly, whereas LLaVA-enhanced CLIP first generates an image-conditioned reformulation (Table 3). Classifier and backbone learning rates, fine-tuning depth, fusion, and imbalance-handling strategies were selected using development data (Table 5 and Table 6). All three final pipelines classify image–tweet pairs using independently L2-normalized modality embeddings. The separate tweet-disjoint Task 3 frozen-encoder study evaluates standalone modalities and a matched CLIP image-only versus image-plus-original-tweet control.

Development-only hyperparameter and model selection.

Hyperparameter selection within a model family precedes task-specific model-family selection. Development-stage tuning varies one group at a time—classifier learning rate, fine-tuning depth, backbone learning rate, weight decay, fusion, and class-imbalance strategy—rather than exhaustively evaluating their Cartesian product. For model family *m*, candidate configuration *c* at a tuning stage, and task t∈{1,2,3}, let $F_{m,c,t}^{\mathrm{DEV}}$ be the macro-F1 measured on that task’s development partition using seed 42, and let $B_{m,c,t}^{\mathrm{DEV}}$ denote balanced accuracy. The cross-task validation summaries are

(10) $${\bar{F}}_{m}^{\mathrm{DEV}}\left(c\right)=\frac{1}{3}\sum_{t=1}^{3}F_{m,c,t}^{\mathrm{DEV}},\;{\bar{B}}_{m}^{\mathrm{DEV}}\left(c\right)=\frac{1}{3}\sum_{t=1}^{3}B_{m,c,t}^{\mathrm{DEV}}.$$

All three tasks contribute equally, rather than being weighted by their unequal numbers of development examples. Candidates are principally compared by ${\bar{F}}_{m}^{\mathrm{DEV}}\left(c\right)$; ${\bar{B}}_{m}^{\mathrm{DEV}}\left(c\right)$ served as a secondary criterion when mean macro-F1 scores are tied or closely similar. Close macro-F1 results are evaluated using the secondary balanced-accuracy summary and manual judgment; no fixed numerical threshold for a near tie is specified in advance. The resulting family-level settings are fixed before the final repeated-seed test evaluations and are stated in Table 6.

For every final training run, the checkpoint epoch is chosen solely by that run’s task-specific development macro-F1, $e_{m,t,s}^{*}=\arg\max_{e}F_{m,t,s,e}^{\mathrm{DEV}}$. With the configurations fixed, the task-wise model-family comparisons are based on mean DEV macro-F1 across the five final seeds, S={42,123,2024,3407,777}:

(11) $${\hat{m}}_{t}=\arg\max_{m}\frac{1}{5}\sum_{s\in S}F_{m,t,s,e_{m,t,s}^{*}}^{\mathrm{DEV}}.$$

This criterion selects CLIP for Tasks 1 and 2 and LLaVA-enhanced CLIP for Task 3. Hyperparameter selection, checkpoint selection, and task-specific model-family selection do not use test-set metrics. The selected configurations are evaluated on the held-out test data at seed 42 and subsequently across the specified five seeds, without test-driven retuning. The final results include all three model families on every task, irrespective of which family ranked first on DEV.

The fusion study holds the pretrained encoder choices and training settings fixed within each model–task comparison, varying only the fusion implementation. For this controlled comparison, all runs use a 336×336 image input, batch size 32, seed 42, weighted cross-entropy, a maximum of 100 epochs, and early-stopping patience 10. The selected fine-tuning depths are five layers for CLIP, three for LLM2CLIP, and seven for LLaVA-enhanced CLIP (applied to both corresponding image and text backbones). The concatenation baseline metrics are retained from matching earlier development runs rather than counted as new independent replications.

The final held-out test experiment uses five seeds (42, 123, 2024, 3407, and 777) for each model and task, after the family-level configuration choices described in Equations (10) and (11) were fixed. The best training epoch is selected by development macro-F1; its checkpoint is then evaluated on the held-out test partition. The test results summarize these five runs by mean and sample standard deviation (sample SD with denominator n−1); the same seed labels identify aligned runs across model families and support seed-blocked comparisons described in Section 4.7. The held-out test results report all four classification metrics for all nine final model–task combinations. Encoder and optimization configurations used for this experiment are provided in Table 6. Fusion is not claimed to improve image–text alignment without separate alignment-specific measurements. The exploratory cross-event study reuses previously selected hyperparameters in a single-seed evaluation (seed 42), with source-event-only grouped development splitting and an entirely held-out target disaster for each test. This additional protocol does not replace the official five-seed benchmark results.

Computational profiling protocol.

Computational requirements are measured for the development-selected configurations across all three tasks (nine model–task runs, seed 42) on an NVIDIA A100-SXM4-40GB (CUDA 12.6, PyTorch 2.9.0). The models use 336×336 RGB inputs and a training batch size of 32. We report total and trainable parameter counts and the parameter/buffer memory footprint, as well as mean train-only seconds per epoch, total training-plus-development wall time, and peak PyTorch-allocated training VRAM. For inference, a fresh process loads the development-selected weights without an optimizer; a separate NVML probe samples process memory every 25 ms during 12 uncached batch-size-one inferences. Latency is the mean of 20 synchronized, warmed-up, uncached test forward passes at batch size one; throughput is computed over the full test split with batches of 32. Inference measurements include the LLaVA frozen image-conditioned text-generation step rather than reusing generated text from a cache. Forward timings exclude image I/O and CPU preprocessing, so they are not application request-to-response latency. PyTorch profiler FLOPs/MACs are retained as partial estimates only because not all operations are covered.

### 4.2. Development-Set Fusion Configuration Comparison

We compare concatenation, global learnable weighted fusion, and feature-wise gated fusion on the development partition for each model–task combination in the fusion-ablation study. The same pretrained model and optimization parameters are used within each comparison. The concatenation conditions use corresponding earlier development evaluations, whereas weighted and gated fusion were evaluated in the subsequent comparison. All values in Table 7 correspond to a single seed (42), not the five-seed final test. The LLaVA fusion comparison uses weighted cross-entropy throughout, whereas the final selected LLaVA configuration uses balanced sampling with ordinary cross-entropy.

At seed 42, concatenation yields the highest observed development macro-F1 in seven of nine model–task comparisons and has the largest unweighted average macro-F1 across the three tasks for each model (82.02% for CLIP, 81.32% for LLM2CLIP, and 80.11% for LLaVA-CLIP). Weighted fusion gives higher observed development macro-F1 than concatenation for CLIP Task 2 (90.08% vs. 89.65%) and LLaVA-CLIP Task 3 (64.42% vs. 63.69%). No gated variant is best in its model–task comparison. These single-seed, development-set findings support concatenation as a simple and competitive configuration choice rather than an intrinsically superior fusion architecture: the ablation uses one seed, and the fusion variants differ in output and downstream MLP input dimensionality. The alternative fusion mechanisms are not subjected to five-seed test evaluation.

### 4.3. Class-Imbalance Strategy Comparison

We examine class-imbalance handling using the same pretrained representations, selected fine-tuning depths, learning rates, weight decay, concatenation fusion, 336×336 images, and batch size 32 within each model–task comparison. The class-imbalance comparison uses the official development partitions and a single training seed (42); no held-out TEST measurements from this comparison are used to choose the strategy. Three loss/sampling choices are evaluated: inverse-training-class-frequency-weighted cross-entropy (weighted CE), class-weighted focal loss with focusing parameter γ=2, and inverse-frequency WeightedRandomSampler (replacement sampling, one epoch comprising the original training-set number of draws) followed by ordinary unweighted cross-entropy (balanced sampler + CE). The weighted-CE reference values come from matching earlier development evaluations; focal loss and balanced sampling are evaluated subsequently. The 27 entries therefore describe distinct model–task–strategy conditions, not 27 independent training-seed replications. Table 8 summarizes the development-set macro-F1 scores for the three class-imbalance strategies.

The highest-scoring imbalance strategy on the single-seed development evaluation varies by model and task. On humanitarian categorization (Task 2), weighted CE gives 89.65% DEV macro-F1 for CLIP, versus 89.80% with weighted focal loss and 89.72% with balanced sampling. On damage assessment (Task 3), CLIP and LLM2CLIP obtain their highest DEV macro-F1 with weighted CE (63.61% and 62.93%), whereas LLaVA-enhanced CLIP obtains 64.17% with balanced sampling, compared with 63.69% for weighted CE. The global model-family selections used for the final five-seed benchmark evaluation (weighted CE for CLIP and LLM2CLIP; balanced sampling with ordinary CE for LLaVA-CLIP) correspond to the largest unweighted three-task average DEV macro-F1 within each family among these candidates. These are development-only, single-seed findings; small differences (such as 0.15 percentage points for CLIP on Task 2) do not establish statistically robust superiority, and no separate five-seed test comparison of the three imbalance strategies was performed.

### 4.4. Controlled Frozen-Representation Component Ablations

For Tasks 1–2, we compare frozen CLIP and LLM2CLIP text representations on the released TRAIN/DEV partitions while holding visual features fixed. The study comprised 60 runs: two tasks, two text encoders, five probe conditions, and three seeds (42, 123, 3407). Cleaned tweets are encoded by either the OpenAI CLIP ViT-L/14-336 text tower or the LLM2Vec/Llama-3 LLM2CLIP text pathway with its pretrained Microsoft adapter, each truncated to 77 tokens using its own tokenizer. All visual features come from the same frozen OpenAI CLIP ViT-L/14-336 encoder. Pretrained features are cached; independently TRAIN-fitted PCA projections reduced each representation to 256 dimensions, followed by L2 normalization. The resulting PCA spaces are not jointly aligned.

The text-only probes used the same classifier structure: LayerNorm, a 256-unit ReLU layer, dropout (0.3), and a task-specific output layer. Concatenation and feature-wise gating project 512 input dimensions to 256 before this classifier, whereas global weighted fusion combines the two 256-dimensional vectors using learned scalar weights. Classifier capacity is matched between CLIP and LLM2CLIP within each probe but not across fusion strategies. All runs use AdamW (learning rate $10^{-3}$; weight decay $10^{-3}$), batch size 32, a 50-epoch limit, patience 10, and DEV macro-F1 for checkpoint selection. Inverse-frequency weighted cross-entropy is the default; the unweighted concatenation control uses ordinary cross-entropy with unchanged minibatch sampling. Table 9 reports mean DEV macro-F1 and sample standard deviation across the three seeds.

The text-only results are nearly identical for Task 1, while CLIP yields the higher mean on Task 2. With shared visual features and the same concatenation classifier, CLIP attains higher DEV macro-F1 on Task 1 (91.72% versus 91.52%), whereas LLM2CLIP is slightly higher on Task 2 (87.68% versus 87.15%). Concatenation yields the highest observed mean among the three weighted-cross-entropy fusion strategies in all four model–task combinations. The unweighted control changes concatenation macro-F1 by between −0.08 and +0.21 percentage points, indicating no consistent gain from class weighting in these probes.

These development-only findings do not establish a superior text representation or fusion architecture. Although visual features and classifier capacity are matched within each probe, the text pathways still differ in tokenizer, pretraining, architecture, adapter, and native dimensionality. Moreover, the frozen 77-token LLM2CLIP probe does not reproduce the final partially fine-tuned 512-token pipeline, and the PCA-fusion predictions do not measure native cross-modal alignment. The observed differences therefore remain conditional on the specified frozen-feature protocol.

We separately evaluate frozen-encoder input conditions for image-derived damage-severity labels using tweet-disjoint Task 3 partitions (2299 TRAIN, 575 DEV, and 529 unchanged TEST pairs). Frozen features are extracted from 336×336 images and from original or image-conditioned enriched tweets. Unused embeddings are zero-masked for single-modality inputs, with an MLP classifier trained for up to 100 epochs (batch size 32; patience 10). The classifier learning rate is selected from $\left\{10^{-4},5\times 10^{-4},10^{-3},2\times 10^{-3}\right\}$ using five-seed mean DEV macro-F1 before TEST evaluation. Table 10 reports three-seed standalone-modality outcomes (42, 123, 3407) and a separately trained, matched five-seed frozen CLIP image-only versus image-plus-original-tweet comparison (42, 123, 2024, 3407, 777).

The standalone-modality results favor image-only input (60.21±0.86% TEST macro-F1) over original tweet text (49.30±3.21%) or image-conditioned enriched text (50.39±0.80%). In the matched five-seed frozen CLIP comparison, adding the original tweet changes mean macro-F1 from 60.99±1.25% to 61.07±1.40%, a difference of +0.08 percentage points. The three-seed subset yields a larger but still small descriptive difference of +0.51 points (image-plus-tweet: 60.73±1.73%). Because enriched text is generated using the paired image, it is not an independent text-only modality. These results neither quantify the effect of text in the final partially fine-tuned pipelines nor establish a general benefit from adding tweet context; no corresponding matched LLM2CLIP Task 3 modality comparison is completed.

### 4.5. Held-Out Test Performance and Class-Level Analysis

Table 11 reports the five-seed held-out results for all model–task combinations. Published CrisisMMD results and their protocol comparability are discussed separately in Section 4.11. Within-study results use identical task-specific test records and label definitions; published results are contextual unless equivalent partitions, inputs, and metric conventions can be established. Published results known to follow incompatible task protocols are excluded from the corresponding fixed-split numerical comparison. The mean and sample standard deviation of macro-F1 are, respectively, 92.32 ± 0.55% for CLIP, 92.39 ± 0.33% for LLM2CLIP, and 91.07 ± 0.30% for LLaVA-enhanced CLIP on Task 1. The corresponding test accuracies are 93.13 ± 0.54%, 93.29 ± 0.30%, and 92.03 ± 0.29%. The near tie between CLIP and LLM2CLIP does not establish an advantage for the LLM-derived text representation. Using aligned seeds, the CLIP minus LLM2CLIP test macro-F1 difference is −0.07 percentage points (paired-seed 95% bootstrap CI [−0.69,+0.56], exact two-sided sign-flip p=0.8750). CLIP exceeds LLaVA-CLIP by +1.26 points (paired CI [+0.88,+1.72]), but its exact paired p=0.0625 and Holm-adjusted p=0.5625 across the full nine within-task contrasts do not meet 0.05. The full seed-blocked statistical analysis is presented in Section 4.7.

For each development-selected model, the following task-specific analyses provide a confusion matrix averaged over five training seeds and an ROC curve for seed 42. All runs within a task use the same fixed test partition; thus, seed-to-seed standard deviations reflect training variability rather than independent test samples.

#### 4.5.1. Informativeness Class-Level Analysis (Task 1)

Task 1 has two categories with different test-set supports: 504 Not informative and 1030 Informative image–tweet pairs. For the development-selected CLIP model, we summarize per-class test precision, recall, and F1 across five training seeds (42, 123, 2024, 3407, and 777). Table 12 presents the arithmetic mean and sample standard deviation of class-wise precision, recall, and F1 across those five independently trained models. The same 1534 test pairs are evaluated in each run; thus variability reflects training-seed changes, not independently sampled test sets.

**Figure 1:**
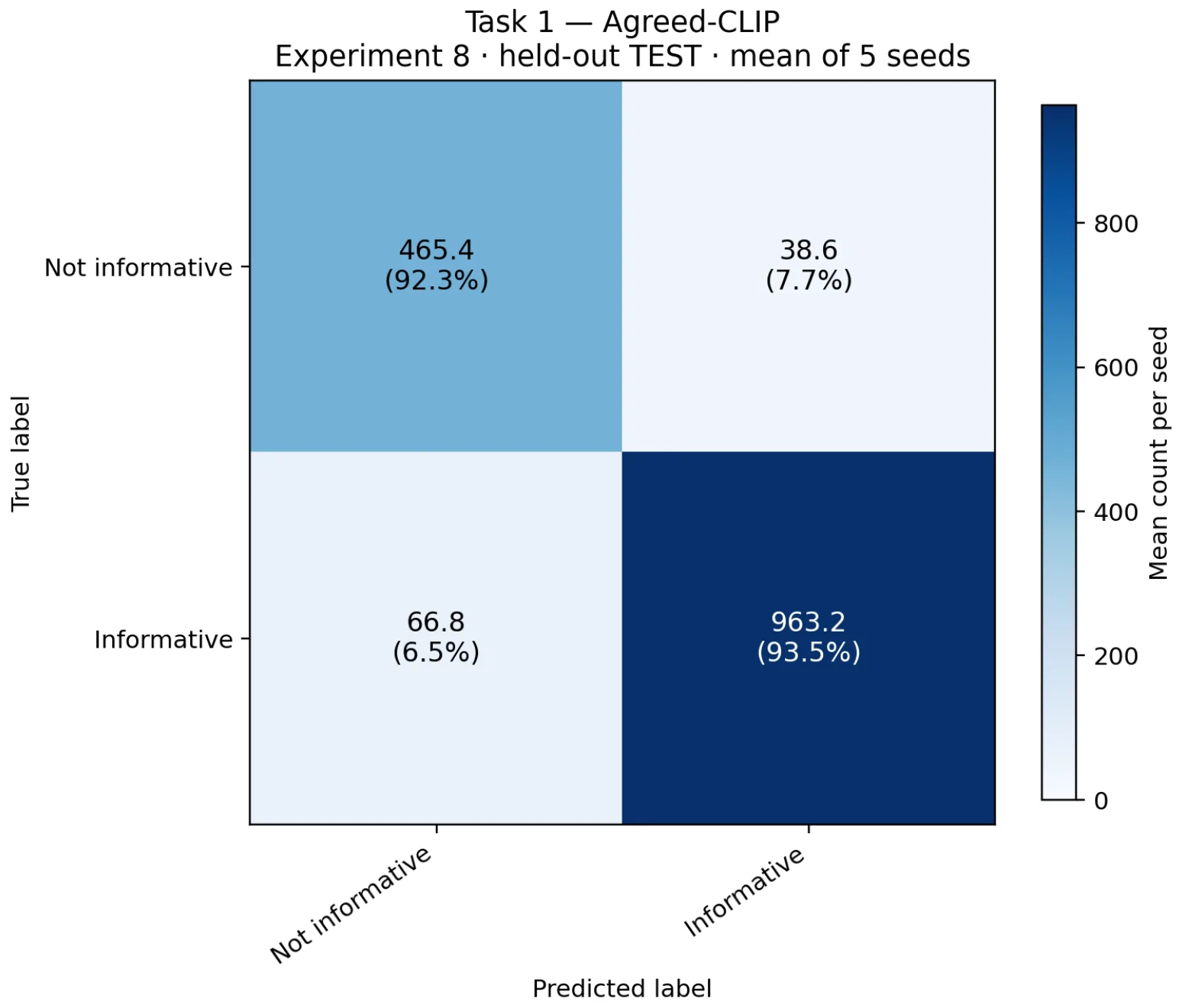
Task 1 CLIP: five-seed mean TEST confusion matrix (n=1534 pairs per seed; row percentages and mean counts).

**Figure 2:**
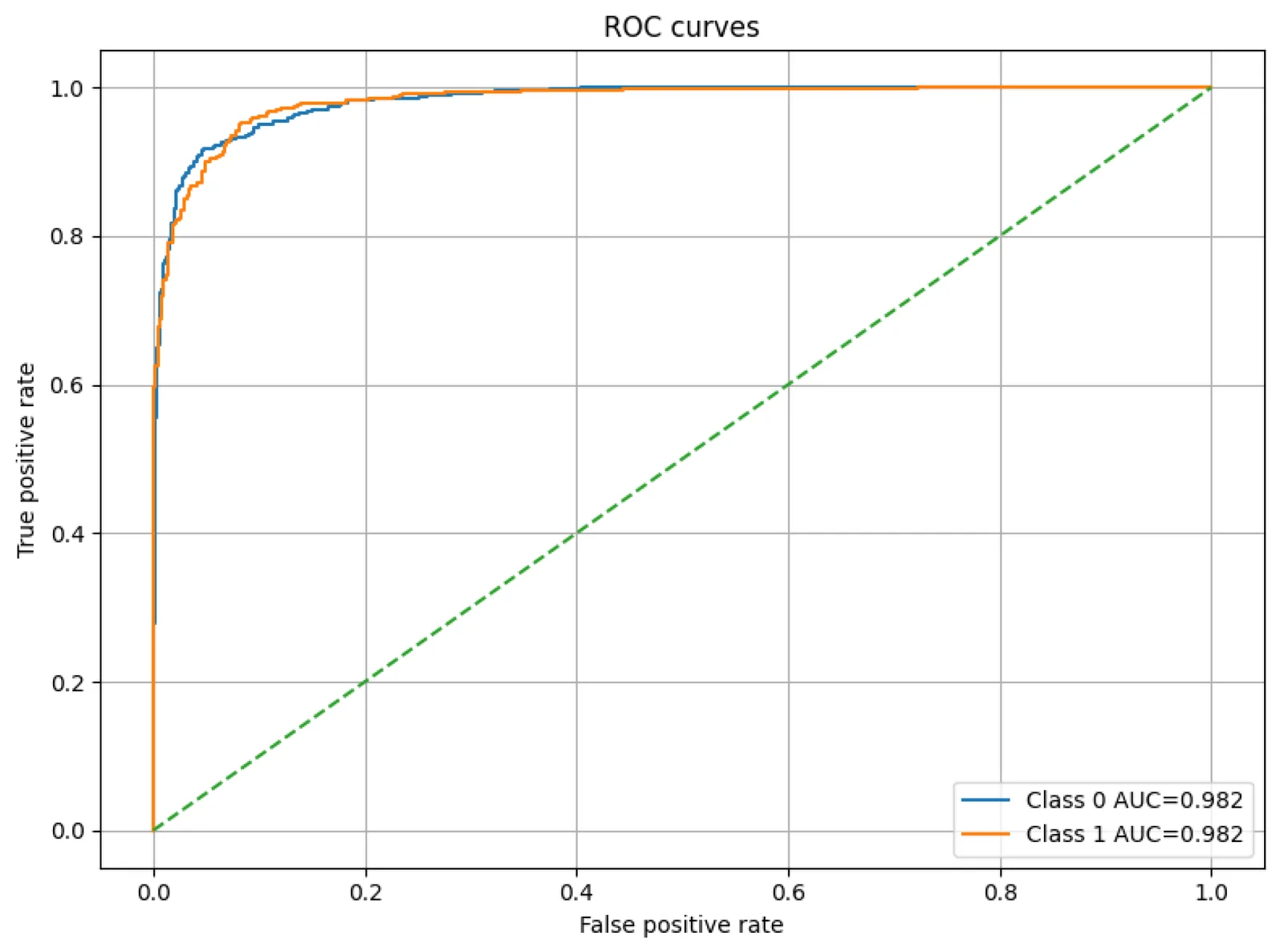
Task 1 CLIP: TEST ROC curve from seed 42 only (not the five-seed mean). The green dashed diagonal indicates chance-level discrimination (true positive rate = false positive rate; AUC = 0.5).

The less frequent Not informative class has lower mean precision (87.50±2.00%) and F1 (89.84±0.65%) than the Informative majority class (precision 96.15±0.48%, F1 94.81±0.45%). Nonetheless, its mean recall (92.34±1.09%) indicates that most non-informative cases in the fixed TEST partition are identified correctly. The five-seed mean confusion matrix in Figure 1 complements these class-level results. The mean of the class-wise F1 values is consistent with the five-seed macro-F1 of 92.32%. Across the same five seeds, one-vs-rest macro ROC-AUC and macro AUPRC are 98.13±0.08% and 97.84±0.09%, respectively; Figure 2 illustrates the ROC curve from seed 42. These estimates characterize performance on the fixed benchmark test partition.

#### 4.5.2. Humanitarian Class-Level Analysis (Task 2)

CLIP is selected for Task 2 using development macro-F1. Its five-seed TEST macro-F1 of 87.52±1.09% exceeds the observed means for LLM2CLIP (83.64±0.69%) and LLaVA-enhanced CLIP (84.44±0.57%), although neither paired comparison reaches significance after Holm correction (see Section 4.7). The following class-level analysis concerns the development-selected CLIP configuration, evaluated across five training seeds on the same 955 image–tweet test pairs. The sample standard deviations therefore describe training variability, not uncertainty across independent test cohorts. Table 13 summarizes the per-class precision, recall, and F1 scores.

**Figure 3:**
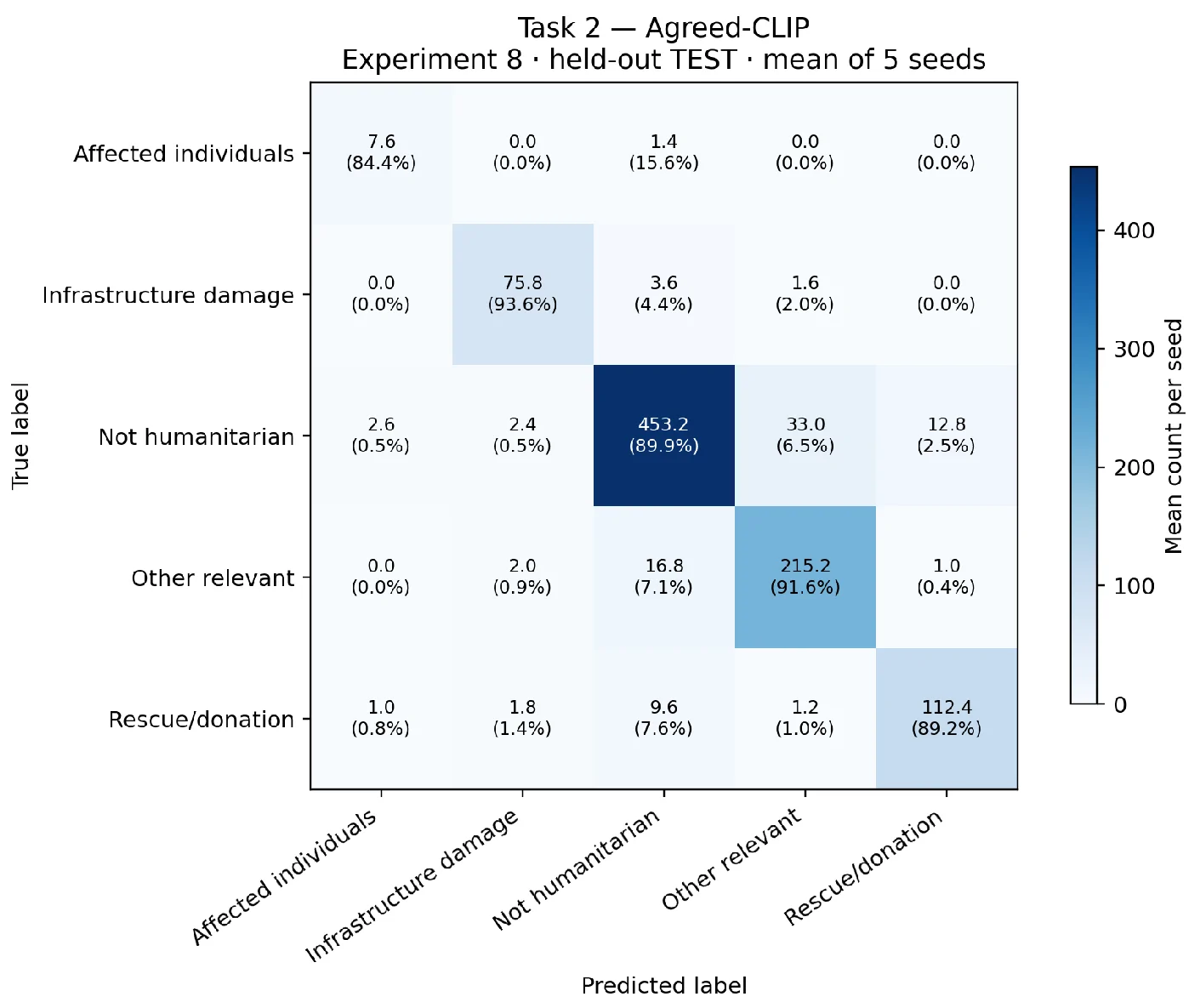
Task 2 CLIP: five-seed mean TEST confusion matrix (n=955 pairs per seed; row percentages and mean counts).

**Figure 4:**
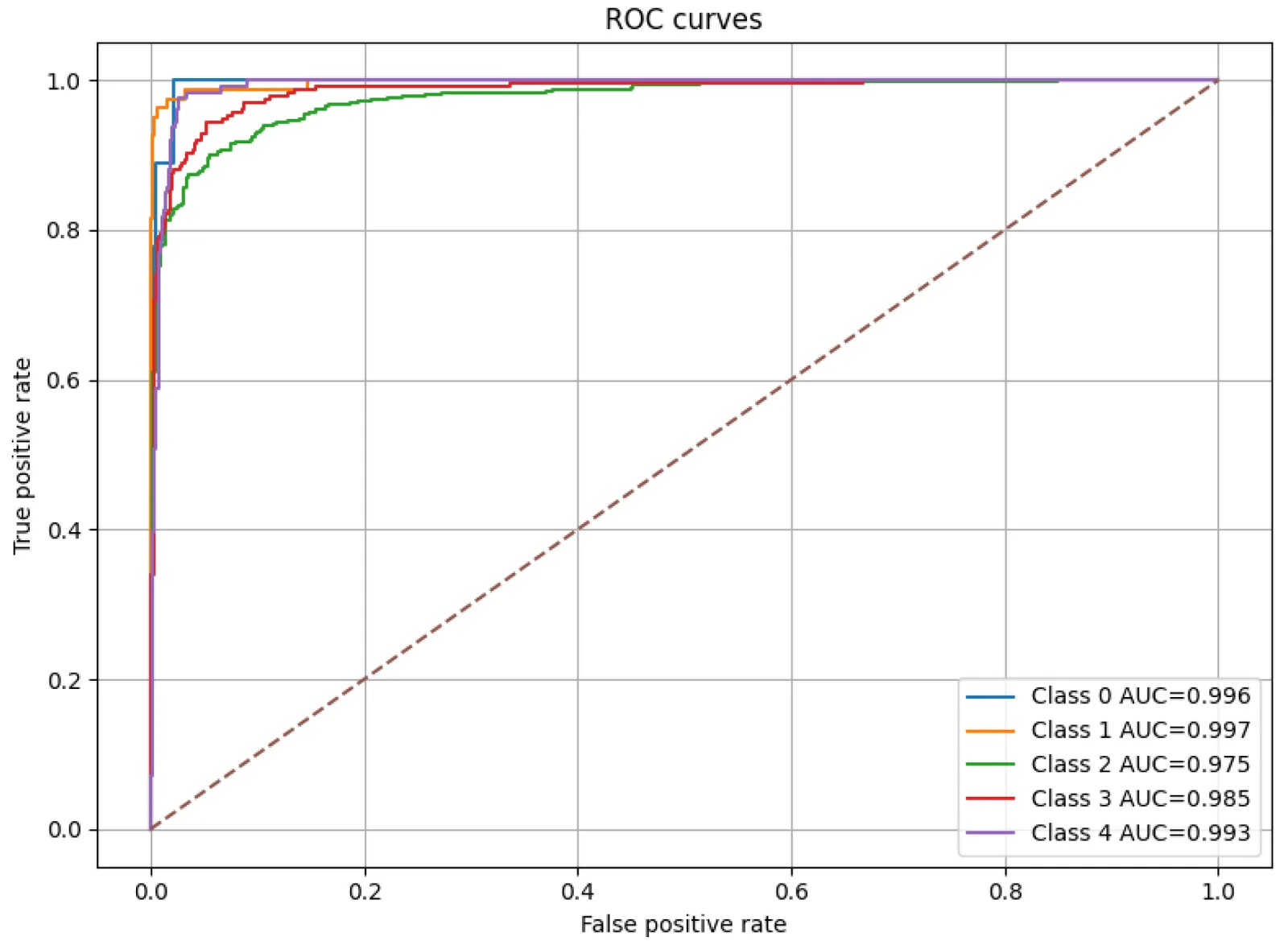
Task 2 CLIP: TEST ROC curve from seed 42 only (not the five-seed mean).

Although the overall accuracy is 90.49%, the Affected individuals class has only nine test pairs and substantially lower precision (67.94%) and F1 (75.21%) than the dominant classes. Thus, its estimates are sensitive to a few individual decisions and should not be treated as robust evidence of performance on underrepresented crisis categories. Balanced accuracy equals macro recall (89.75±1.96%); five-seed one-vs-rest macro ROC-AUC and macro AUPRC are 98.86±0.05% and 93.67±0.28%, respectively. Figure 3 shows the five-seed mean confusion matrix, with confusion concentrated particularly between Not humanitarian and Other relevant information. Figure 4 provides the ROC visualization for seed 42. These aggregate discrimination and ranking measures do not replace threshold-dependent per-class performance or confusion-matrix inspection. We do not use micro-average ROC-AUC as the primary indicator of performance for this imbalanced five-class task.

#### 4.5.3. Damage-Severity Class-Level Analysis (Task 3)

The development-selected Task 3 model is LLaVA-enhanced CLIP with inverse-frequency balanced sampling and ordinary cross-entropy. Its class-level test results are aggregated across five training seeds (42, 123, 2024, 3407, and 777), each evaluated on the same 529 image–tweet pairs. Little/no damage has 71 test examples, mild damage has 126, and severe damage has 332. Table 14 gives class-wise mean precision, recall, and F1 with sample standard deviations across training seeds.

**Figure 5:**
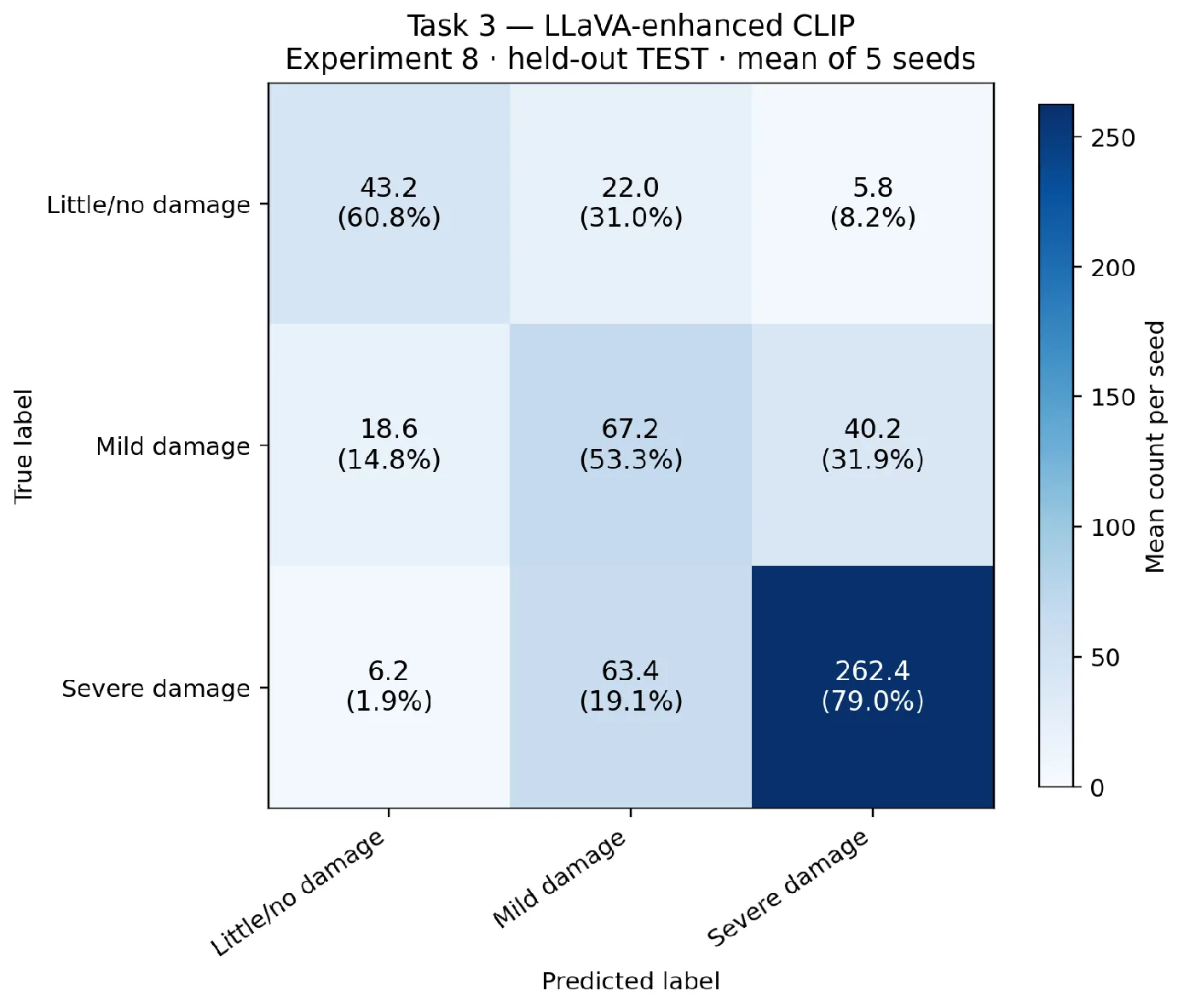
Task 3 LLaVA-enhanced CLIP: five-seed mean TEST confusion matrix (n=529 pairs per seed; row percentages and mean counts).

**Figure 6:**
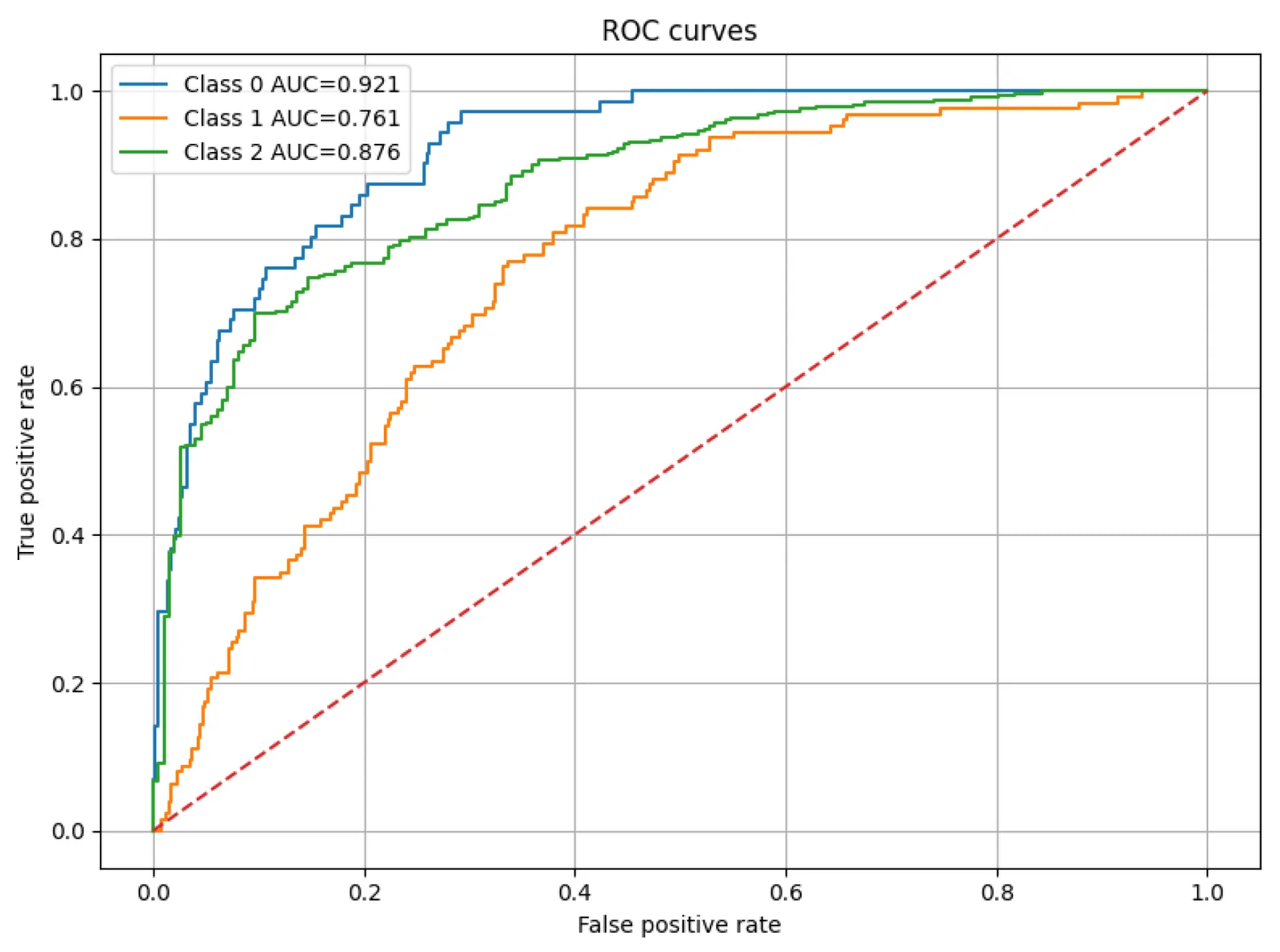
Task 3 LLaVA-enhanced CLIP: TEST ROC curve from seed 42 only (not the five-seed mean). The red dashed diagonal indicates chance-level discrimination (true positive rate = false positive rate; AUC = 0.5).

The mild damage class has the weakest F1 (48.17 ± 2.48%) and precision (43.99 ± 1.00%), despite having more test samples than the little/no-damage class. The dominant severe-damage class achieves substantially higher F1 (81.95±0.29%). The five-seed mean confusion matrix (Figure 5) provides the corresponding error distribution. Across five seeds, the development-selected LLaVA-enhanced CLIP model attains one-vs-rest macro ROC-AUC of 85.15±0.25% and macro AUPRC of 67.85±0.27%; Figure 6 shows the ROC curves for seed 42. Neither these aggregate ranking measures nor the overall TEST accuracy (70.47±0.47%) and macro-F1 (64.06±0.98%) establish uniformly reliable severity recognition across classes. This minority-class assessment describes the chosen LLaVA pipeline, not an independently established causal effect of balancing. The class-level estimates come from one fixed benchmark test partition, so they characterize run-to-run variation rather than performance across new disaster populations. The benchmark Task 3 results and figures above refer exclusively to the released, partially tweet-overlapping split; the retrained tweet-disjoint results, with separate tables and figures, appear in Section 4.6.

### 4.6. Tweet-Disjoint Damage-Severity Leakage Sensitivity Study

Motivation and split construction.

Our audit identifies 62 tweet identifiers shared by the official Task 3 training and test sets, as well as repeated tweet identifiers between training/development and development/test. Since the classifiers use paired tweet text, distinct image IDs alone do not ensure text-independent evaluation. We therefore conduct a Tweet-Disjoint Damage-Severity Leakage Sensitivity Study that reconstructs group-disjoint training and development partitions before model fitting and checkpoint selection. We retain all 529 official TEST image–tweet pairs unchanged (71 little/no, 126 mild, and 332 severe damage). Starting from the 2997 released TRAIN and DEV pairs, we remove 123 records whose tweet identifier occurs in the official TEST partition, leaving 2874 eligible records. A fixed five-fold stratified tweet-group split (seed 42; frozen first-fold group membership) yields 2299 TRAIN and 575 DEV pairs, with TEST remaining at 529 pairs (total retained: 3403). The resulting class counts are shown in Table 15. The split manifest records source-file fingerprints and the fixed development tweet groups, making the partition reproducible across software environments.

Leakage checks and controlled retraining.

The reconstructed partitions have zero cross-split intersections in tweet IDs, image IDs, image paths, and exact or normalized tweet text. These checks rely on metadata and text, not image-byte hashing or perceptual near-duplicate detection. All three pipelines are retrained from their respective pretrained checkpoints on the same 2299-pair TRAIN set; checkpoints are selected by macro-F1 on the 575-pair tweet-disjoint DEV set and evaluated on the unchanged 529-pair TEST set. Each model retains its architecture and optimization settings selected under the released benchmark protocol, including fine-tuning depth and class-imbalance strategy. Five seeds (42, 123, 2024, 3407, and 777) are evaluated per model, giving 15 runs. TEST scores are not used for checkpoint or hyperparameter selection. Because the fixed configurations originate from the released development protocol, this sensitivity study does not constitute fully independent model selection.

Performance under tweet-disjoint evaluation.

**Figure 7:**
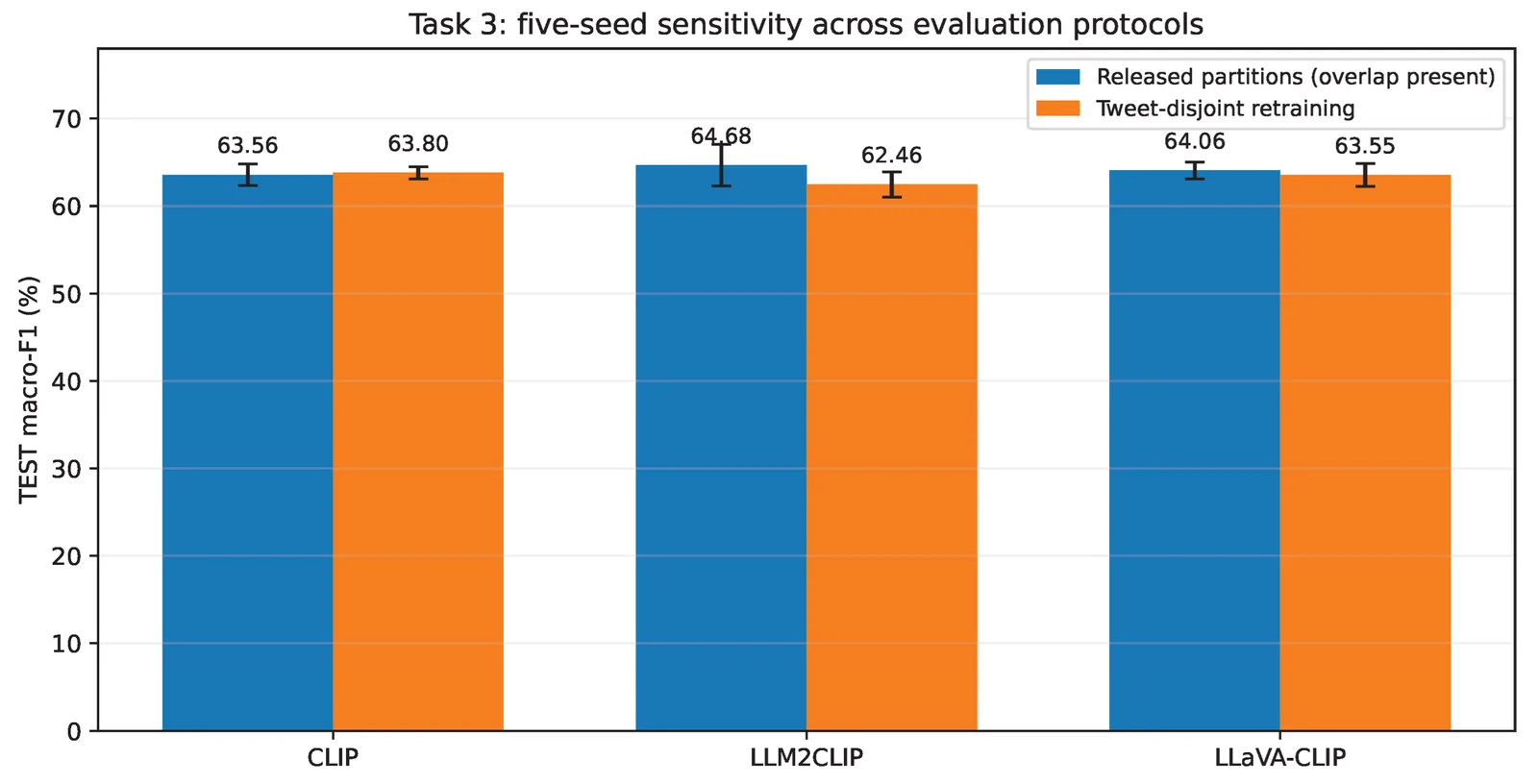
Task 3 TEST macro-F1 mean and sample standard deviation across five training seeds. Bars compare the original released-partition models with models independently retrained after removing cross-split tweet-identifier overlap. The fixed test records are the same; TRAIN/DEV membership differs, so differences cannot be assigned exclusively to leakage. Both bars per model use image–tweet input; no image-only comparison is shown.

Table 16 compares macro-F1, accuracy, and balanced accuracy under the original and tweet-disjoint protocols, each evaluated across five seeds on the same 529 TEST pairs. Mean macro-F1 changes by +0.24, −2.22, and −0.51 percentage points for CLIP, LLM2CLIP, and LLaVA-enhanced CLIP, respectively, altering their descriptive ranking (Figure 7). Accuracy and balanced accuracy do not necessarily change in the same direction: for LLaVA-enhanced CLIP, accuracy increases by 0.87 points while balanced accuracy decreases by 1.35 points. Damage-severity predictive performance persists under tweet-disjoint retraining, but differences between protocols cannot be attributed exclusively to the removal of repeated tweet identifiers because training exposure and development-set selection also change; statistical equivalence is neither tested nor claimed.

Class-level behavior and diagnostic plots.

**Figure 8:**
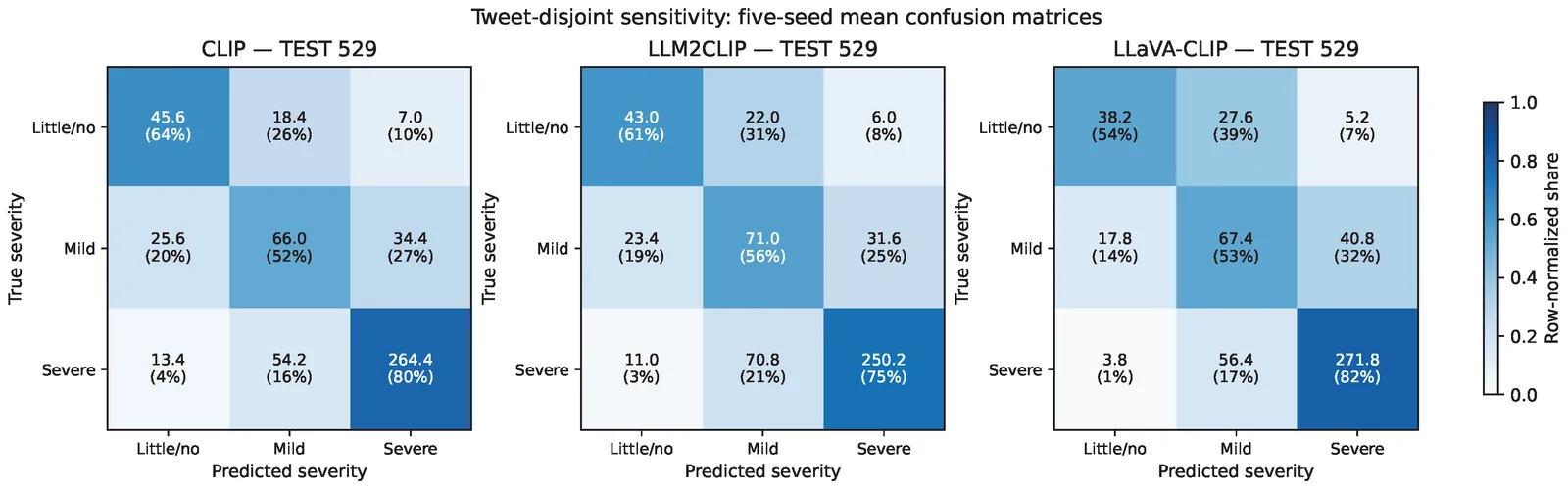
Tweet-disjoint Task 3: five-seed mean TEST confusion matrices for CLIP, LLM2CLIP and LLaVA-enhanced CLIP, each on the unchanged 529-pair TEST set. Counts are means across models’ five seed-specific matrices, not counts from additional independent TEST pairs; percentages are row-normalized.

**Figure 9:**
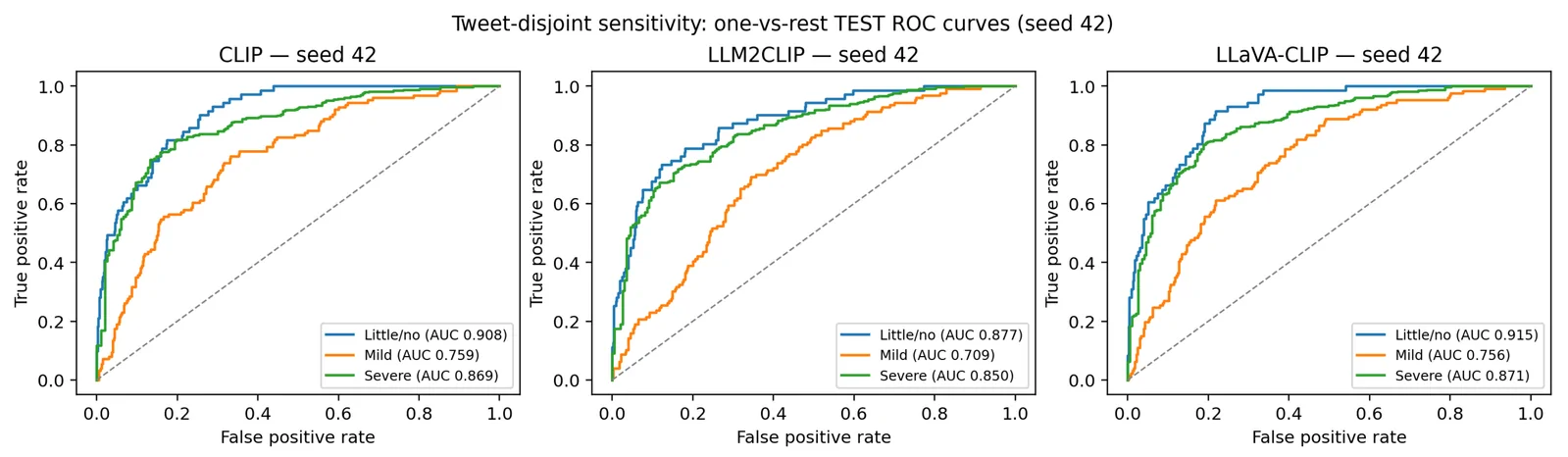
Tweet-disjoint Task 3 one-vs-rest TEST ROC curves for the three model families, evaluated at seed 42 only on the unchanged 529 TEST pairs; legend AUCs are per-class seed-42 values, not five-seed estimates. The dashed diagonal reference lines indicate chance-level discrimination (true positive rate = false positive rate; AUC = 0.5).

Table 17 reports class-wise TEST F1. Intermediate mild damage remains the most difficult class for each pipeline (48.58–49.88% mean F1), while severe damage yields the largest class F1 (80.69–83.66%). The five-seed mean confusion matrices in Figure 8 show mean cell counts and row-normalized percentages. For visual completeness, Figure 9 presents one-vs-rest TEST ROC curves for seed 42 only; these curves must not be read as five-seed averages.

Interpretation and residual limits.

The tweet-disjoint study removes observed tweet-identifier overlap across supervised training, checkpoint selection, and TEST evaluation while preserving the released TEST records. It remains a sensitivity analysis: the training and development records differ from those in the original benchmark, the configurations are selected under the released protocol, and metadata checks cannot exclude paraphrased tweets or visually near-duplicate images. The observed class-dependent performance therefore does not isolate the effect of repeated tweet IDs or establish significant model-family differences. Both retraining protocols use image–tweet input; the separate frozen-encoder modality and matched CLIP controls in Table 10 address the contribution of tweet text under their own experimental conditions.

### 4.7. Multi-Seed Uncertainty and Seed-Blocked Statistical Comparisons (Tasks 1–3)

The 45 original-benchmark TEST evaluations comprise three model families, three tasks, and five random seeds (42, 123, 2024, 3407, and 777). Development macro-F1 selected CLIP for Tasks 1 and 2 and LLaVA-CLIP for Task 3, independently of TEST outcomes (Equation (11)). Within each task, all models are evaluated on the same fixed TEST records using corresponding seed labels. Statistical comparisons therefore use matched training seeds as blocks, preserving the paired experimental structure.

For a model pair (A,B) in task *t*, we compute the five within-seed test macro-F1 differences in percentage points,

(12) $$d_{t,s}\left(A,B\right)=100\left[F_{A,t,s}^{\mathrm{TEST}}-F_{B,t,s}^{\mathrm{TEST}}\right],\;{\bar{d}}_{t}=\frac{1}{5}\sum_{s\in \{42,123,2024,3407,777\}}d_{t,s}\left(A,B\right).$$

We apply a two-sided exact paired sign-flip randomization test to the observed mean difference, enumerating all $2^{5}=32$ independent within-block sign assignments under a sign-exchangeability null. The *p*-value is the fraction of sign assignments with absolute mean difference at least as large as observed; no Monte Carlo approximation is needed. We calculate descriptive, unadjusted 95% paired percentile-bootstrap intervals by resampling the five seed blocks together for the two model families with replacement; all $5^{5}=3125$ possible ordered resamples are enumerated. The bootstrap resampling therefore preserves the pairing, rather than sampling each model independently. These percentile intervals are not multiplicity-adjusted, can differ in inference from the discrete exact test, and are not claimed to provide exact small-sample coverage.

Because no model-family superiority hypothesis is established a priori across the three tasks, we define one exploratory multiple-testing family comprising all nine within-task pairwise contrasts: three model pairs for each of the three tasks. We apply Holm’s step-down adjustment jointly to the nine exact paired *p*-values. The unadjusted *p*-values and Holm-adjusted results appear in Table 18. With only five paired blocks, the smallest possible two-sided exact sign-flip *p*-value is 2/32=0.0625. Thus these data cannot cross an unadjusted 0.05 threshold under this exact test, regardless of effect magnitude; statistical power is necessarily limited. Neither significance nor equivalence can be established from the non-rejections.

None of the nine within-task model comparisons is statistically significant under the exact seed-paired tests after Holm adjustment (minimum adjusted p=0.5625). Descriptively, CLIP exceeds LLaVA-CLIP on Task 1 by +1.26 macro-F1 points (unadjusted paired 95% interval [+0.88,+1.72]), and on Task 2 exceeds LLM2CLIP by +3.88 points ([+3.16,+4.50]) and LLaVA-CLIP by +3.08 points ([+2.09,+4.06]). All five paired seeds favor CLIP in these three contrasts, but each has exact two-sided p=0.0625 and Holm-adjusted p=0.5625. These observations indicate task-specific descriptive differences, not statistically established superiority by one pretrained pipeline family.

A paired percentile-bootstrap interval may exclude zero even when the exact sign-flip test is non-significant because the procedures have different finite-sample behavior and the $2^{5}$ exact test is especially discrete at n=5. We therefore prioritize the exact, multiplicity-adjusted paired tests for significance statements and use intervals to describe effect direction and magnitude only. These comparisons are conditional on the same fixed TEST examples and five training-seed blocks, not independent re-samples of disaster events; pairing by seed also does not guarantee identical optimization trajectories across different architectures. The Task 3 tweet-disjoint sensitivity study (Section 4.6) uses a separate evaluation protocol.

### 4.8. Exploratory Cross-Event Transfer

To assess transfer to a disaster absent from supervised training and development, we conduct 27 additional event-held-out evaluations (three pretrained model pipelines × three tasks × three target disasters) using the selected configurations from the final five-seed benchmark evaluation and one training seed (42). We select Hurricane Harvey, Mexico Earthquake, and California Wildfires as three contrasting disaster contexts; the remaining four events were not individually held out. For each task and target event, all rows belonging to that event in the released CrisisMMD train, development, and test TSVs are pooled solely for the unseen-event test. The training/validation pool comprises the original training and development records from the other six events; their original test records are not used. An approximately 80/20 StratifiedGroupKFold partition (K=5, fold 0, split seed 42), grouped by tweet_id and stratified by class, supplies the source-event training and development samples. Checkpoint selection remains based on source-event development macro-F1. The resulting partitions contain no shared tweet IDs, image IDs, or exact tweet texts across training, development, and test. All three model pipelines use the same target records for each task–event combination.

As shown in Table 19, informativeness classification (Task 1) transfers comparatively well across the three target events, with macro-F1 ranging from 84.71% to 92.82% across model–event combinations. Humanitarian categorization (Task 2) is more variable (63.33–79.98% macro-F1), and no model is best on every target event. In particular, the Affected individuals class contains only 21, 9, and 8 test pairs for Hurricane Harvey, Mexico Earthquake, and California Wildfires, respectively, limiting confidence in individual minority-class estimates. Damage-severity assessment (Task 3) shows the weakest and most heterogeneous transfer (36.09–58.08% macro-F1). On Mexico Earthquake, CLIP obtains 80.34% accuracy but only 36.49% macro-F1 and 36.64% balanced accuracy, consistent with the pronounced event-specific class imbalance (5 little/no-damage, 25 mild-damage, and 148 severe-damage pairs). These differences should not be interpreted as statistically established model rankings.

The cross-event analysis is exploratory: only three of the seven disasters are held out individually, and each model–task–event combination is run once. In addition, although the held-out target event is excluded from the supervised TRAIN and DEV partitions of the event-held-out analysis, the fixed configurations are selected previously using mixed-event benchmark development data. The evaluation therefore assesses supervised event-held-out transfer under those fixed choices, not fully event-blind hyperparameter optimization. The cross-event test populations also differ from the original benchmark test sets, so Table 11 and Table 19 are not paired comparisons. Evidence from one dataset and three selected disasters cannot establish robustness to arbitrary unseen disasters or other data sources.

### 4.9. Cross-Modal Alignment and Image–Text Retrieval

To assess whether changes in supervised classification performance correspond to measurable image–text embedding alignment, we conduct held-out test analyses separate from the classification metrics. The shared-protocol alignment analysis evaluates CLIP and LLM2CLIP using a common single-seed (42) training protocol (three fine-tuned transformer blocks, concatenation, and class-weighted cross-entropy). A separate text-enrichment analysis evaluates LLaVA-enhanced CLIP under its independently selected final configuration (seven CLIP blocks fine-tuned, concatenation, balanced sampling with cross-entropy; seed 42). All evaluations use the released, unchanged task-specific TEST pairs: n=1534 (Task 1), 955 (Task 2), and 529 (Task 3). The evaluated image and text representations are the individually $\ell_{2}$-normalized encoder outputs in their respective models’ native joint embedding spaces; no additional alignment projection or contrastive optimization is introduced for these experiments.

For normalized image features $v_{i}$ and text features $t_{i}$, the matched-pair similarity is $s_{i}^{+}=v_{i}^{T}t_{i}$. The mismatched baseline is estimated using ten seeded, nonidentity random permutations $\pi_{r}$ of test text indices, and alignment separation is defined by

(13) $$\Delta_{\mathrm{align}}=\frac{1}{N}\sum_{i=1}^{N}s_{i}^{+}-\frac{1}{10N}\sum_{r=1}^{10}\sum_{i=1}^{N}v_{i}^{T}t_{\pi_{r}\left(i\right)}.$$

We additionally rank all *N* candidate texts by image–text cosine similarity and report image-to-text Recall@*K* (K∈{1,5,10}). The primary retrieval protocol considers the paired text at the same dataset index as the only positive, matching the shared-protocol alignment analysis. This is a strict index-level retrieval definition: repeated source tweets can yield other semantically valid candidates, especially in Task 3, so the reported values are not exhaustive measures of semantic retrieval correctness. The retrieval metrics therefore characterize the discrimination of dataset-index pairings under this definition; they do not constitute a manual audit of whether the tweet content accurately describes the damage visible in each image. A supplementary LLaVA analysis also examines retrieval with shared source tweets treated as related candidates; these values are kept separate from the primary index-level retrieval results.

Table 20 shows that LLM2CLIP has a higher matched-pair cosine than CLIP on every task, but it also assigns higher similarity to mismatched pairs. Consequently, CLIP obtains a larger matched–mismatched gap and higher Recall@1, Recall@5, and Recall@10 on all three tasks in the shared-protocol CLIP–LLM2CLIP comparison. These direct diagnostics do not corroborate an unqualified claim that the evaluated LLM2CLIP system improves cross-modal semantic alignment over the CLIP comparator. In Task 3, repeated tweet texts also complicate strict one-positive-per-image retrieval; the measured gaps and recalls must not be interpreted as proof that source tweets are generally misaligned with their paired images. The model families also have different pretrained visual checkpoints and different embedding dimensions, so isolated cosine magnitudes cannot identify the contribution of LLM-derived text semantics.

The LLaVA text-enrichment analysis additionally provides a controlled within-model text-input comparison: the same restored, development-selected LLaVA-enhanced CLIP checkpoint encodes each paired image together with either the cleaned original tweet or the image-conditioned LLaVA rewrite. The visual weights, text weights, and test pairs are unchanged, and no additional gradient updates are made. Table 21 summarizes the differences in strict index-level retrieval and separation.

All three tasks show positive descriptive changes in alignment gap and Recall@*K* after LLaVA rewriting, a pattern partly expected because the generator receives the paired evaluation image as input. This suggests that the resulting text is more consistent with the paired image in the trained image-conditioned rewriting pipeline. It does not establish intrinsic alignment improvement between independently acquired modalities: LLaVA directly observes the evaluation image when producing the enriched text, and the CLIP towers were fine-tuned using such rewrites. The original-text comparison is therefore a post hoc input-control diagnostic, not an independently retrained no-enrichment ablation or a causal demonstration that generation improves downstream classification. Both analyses use one training seed and a fixed benchmark test set; without repeated runs and corresponding uncertainty estimates, small differences should not be treated as statistically established. We therefore restrict our mechanism-level conclusions to the measured similarity separation and retrieval behavior, rather than claiming universal cross-modal alignment superiority.

### 4.10. Computational Cost and Deployment Feasibility

The selected CLIP, LLM2CLIP, and LLaVA-enhanced CLIP pipelines were profiled on a common A100 GPU at seed 42. Table 22 reports parameter counts and model/buffer footprints; Table 23 summarizes single-sample latency, batch throughput, peak GPU memory, and training time across all three tasks. The uncached inference protocol includes LLaVA-based image-conditioned text generation, allowing the observed computational costs to be attributed to the evaluated pipelines rather than inferred from encoder parameter counts alone.

CLIP has the lowest warmed-up batch-size-one inference latency (34.18–34.25 ms) and inference process memory (2.20 GiB). In comparison, LLM2CLIP takes 81.70–84.18 ms and 15.73 GiB, while LLaVA-CLIP requires 1189.68–1314.39 ms and 15.23–15.32 GiB because its image-conditioned LLaVA generator must produce text before classification. Conversely, LLM2CLIP has the highest measured batch-32 throughput (140.99–145.53 samples/s), compared with approximately 42.44–42.51 for CLIP and 0.86–0.92 for LLaVA-CLIP. Throughput rankings at BS = 32 do not imply lower latency at BS = 1 or superior general scalability. The memory and latency measurements indicate that the LLM-based pipelines are computationally demanding under the evaluated conditions.

The training peak is not interchangeable with inference peak: the former is PyTorch-allocated optimizer/training memory, whereas the latter is sampled full-process NVML memory in a separate inference-only run. In particular, the frozen LLaVA generator is removed during downstream CLIP-classifier training; its small reported training peak must not be interpreted as the full memory requirement for uncached inference or for offline generation of augmented text. Moreover, the reported epoch and training times depend on early stopping and task sample counts; they do not incorporate the full cost of preparing the LLaVA augmented text. Model/buffer size is an in-memory footprint, not a measured serialized deployment artifact. The experimental comparison with CLIP is hardware controlled, but literature baselines were not independently profiled in this environment, so we cannot claim a hardware-matched efficiency advantage over other published systems.

Table 24 reports the profiler-supported portion of the FLOPs/MACs estimates. PyTorch’s supported-operator accounting does not yield reliable full-graph totals for all encoder and generation operations, and LLaVA token generation is input dependent; these values must not be used as exact cross-model compute totals.

Despite the favorable forward latency of CLIP on this GPU, the measurements exclude dataset ingestion, image decoding, network transport, queuing, CPU preprocessing, and service orchestration, and they contain no concurrency/load tests. We therefore do not claim measured near-real-time application latency, production scalability, or deployment readiness for any model. The reported cost/performance trade-offs describe the specified hardware and batch sizes only.

### 4.11. Protocol Comparability of Published Baselines

Published CrisisMMD studies differ in their use of released versus study-specific partitions, label mappings, input modalities, supervision, and evaluation metrics. Table 25 distinguishes studies that explicitly adopt the released agreed-label benchmark from those with documented protocol differences and those for which partition identity cannot be established. Matching released partitions establishes a stronger basis for contextual comparison than dataset name alone; it does not control model training or metric averaging, nor does it independently verify record IDs in a third-party implementation. The three pipelines evaluated here use identical task-specific records and targets. Separately, the image-only CLIP evaluation in [25] uses event-specific conditions and weighted-F1 rather than our paired multimodal macro-F1 protocol.

Several published studies document use of the released agreed-label Tasks 1–2 evaluation splits, including BERT + RegNetY320, FixMatchLS (in its official-split experiment), BERT-LSTM + ResNet50, LLaVA + RoBERTa, and Multihead APM. Their reported results can be compared at the level of the published benchmark, subject to differences in architectures, training data, preprocessing, and F1 averaging. In particular, FixMatchLS and Multihead APM use additional unlabeled examples absent from our supervised protocol. By contrast, VGG16 + FastText and ViT-16 + GPT-2+RF use different partitions, while the DMCC humanitarian task defines eight classes rather than our five. For studies without sufficiently detailed split information, exact partition equivalence remains unresolved. The only fully controlled, same-input comparisons are among our three evaluated pipelines. External values are reproduced from the respective publications, not independently rerun, and are not used to infer statistically established superiority.

Table 26 reports studies with documented differences in partitioning or class definitions. These results are separated from Table 27 because identical test populations or prediction targets cannot be assumed.

## 5. Discussion

### 5.1. Task-Dependent Classification Performance

The evaluated LLM-derived and image-conditioned text pipelines do not show a consistent classification advantage over CLIP across the three tasks (Table 11). CLIP and LLM2CLIP obtain similar five-seed informativeness macro-F1 (92.32±0.55% and 92.39±0.33%, respectively), whereas CLIP has the highest observed humanitarian macro-F1 (87.52±1.09%). These are comparisons of complete pipelines with different pretrained components and selected adaptation settings; they do not identify the isolated contribution of language-model-derived features. Nor do the findings imply that larger language models are generally ineffective for crisis classification.

For damage-severity classification, LLM2CLIP has the highest observed mean macro-F1 (64.68±2.37%), while LLaVA-enhanced CLIP (64.06±0.98%) was selected by development performance. The LLM2CLIP–CLIP mean difference of +1.12 points has an exact paired p=0.2500 and an unadjusted 95% paired bootstrap interval of [−0.40,+2.67] points. Across all nine within-task model contrasts, none is significant after Holm correction (minimum adjusted p=0.5625; Table 18). The tweet-disjoint sensitivity study further changes the descriptive Task 3 ranking, reinforcing the need to interpret pipeline comparisons as protocol-dependent rather than statistically established superiority.

The separate tweet-disjoint frozen-encoder controls clarify the role of input modality for image-derived damage labels. Original tweet-only and image-conditioned enriched-text-only models have lower macro-F1 than the image-only model, while adding original tweet text to a matched frozen CLIP image classifier changes five-seed macro-F1 by just +0.08 points (Table 10). These findings do not quantify the contribution of text in the final partially fine-tuned pipelines. Because severity is annotated at image level and enriched text is generated from the image itself, lower standalone text performance cannot be interpreted as a direct measure of intrinsic image–tweet semantic alignment.

### 5.2. Fusion, Representation, and Class-Imbalance Effects

In the single-seed development-set comparison, concatenation achieves the highest observed macro-F1 in seven of nine model–task combinations, while global weighted fusion leads in the remaining two; feature-wise gating does not lead in any comparison (Table 7). These findings support concatenation as a competitive configuration choice under the evaluated protocol but do not establish statistical or capacity-controlled superiority. The fusion alternatives differ in downstream classifier input dimensions and parameter counts, and the experiments concern pooled-feature fusion rather than token-level cross-attention systems such as those of Abavisani et al. [23] and Li et al. [26].

The controlled frozen-representation experiments provide a complementary perspective on the text encoders. Using the same frozen CLIP image features and matched downstream classifier capacity within each probe, neither the text-only nor multimodal comparisons show a consistent advantage for LLM2CLIP-derived text features across Tasks 1 and 2 (Table 9). For example, in the multimodal concatenation probe, LLM2CLIP text features slightly exceed CLIP text features on Task 2, but CLIP features are higher on Task 1. This result narrows the possible interpretation of the final model-family differences; it does not isolate language-model capacity from tokenizer, pretraining, adapter, or architecture effects, and the frozen 77-token probe is not equivalent to the final partially fine-tuned 512-token LLM2CLIP pipeline. The matched unweighted-cross-entropy control in this study likewise does not show a uniform benefit from class weighting. Table 9 contains DEV-only probes, whereas Table 10 reports TEST outcomes from the separately DEV-selected, tweet-disjoint Task 3 controls. Their differing encoders, feature preprocessing, and evaluation partitions must not be conflated.

Class-sensitive evaluation reveals weaknesses that aggregate accuracy obscures. For Task 1, the Not informative class has lower mean test F1 (89.84%) than Informative (94.81%) (Table 12). In Task 2, the development-selected CLIP classifier reaches 90.49% accuracy but 87.52% macro-F1, and the Affected individuals category, represented by only nine test pairs, has a mean F1 of 75.21% (Table 11 and Table 13). In Task 3, Mild damage has the lowest mean F1 (48.17%) despite having more test examples than Little or no damage (Table 14). These observations indicate that class frequency alone does not account for all classification errors, and that macro-averaged and class-level measures are necessary alongside accuracy and ROC-based measures. The single-seed development comparison informs the model-family-specific imbalance-handling choices but does not establish superiority of weighted cross-entropy, focal loss, or balanced sampling (Table 8).

### 5.3. Cross-Modal Alignment and Cross-Event Transfer

The embedding-space results do not support inferring improved image–text semantic alignment from the classification performance of LLM2CLIP. Under the shared-protocol, single-seed comparison, CLIP produces larger matched-minus-mismatched cosine-similarity gaps and higher image-to-text Recall@1, Recall@5, and Recall@10 than LLM2CLIP on all three tasks (Table 20). LLM2CLIP’s larger absolute matched-pair cosine similarities are accompanied by larger mismatched-pair similarities, so absolute cosine magnitude alone is not evidence of stronger alignment. Because the pretrained model families also differ in their visual checkpoints, these observations do not isolate the influence of LLM-derived linguistic representations.

Within the LLaVA-enhanced CLIP pipeline, replacing the original tweet with the image-conditioned rewrite increases the similarity gap and retrieval recalls when evaluated using the same trained downstream CLIP weights (Table 21). The gains describe the behavior of the evaluated rewriting pipeline, not an improvement in the intrinsic alignment of independently supplied image and text modalities: the LLaVA generator observes the retrieval image, and the classifier is trained using enriched text. This single-seed input comparison is also not a matched, independently retrained no-enrichment classification ablation. Consequently, neither alignment analysis establishes a general causal mechanism for the observed task-level performance differences.

The event-held-out evaluation further indicates that predictive behavior varies across disaster contexts. Informativeness classification yields macro-F1 values of 84.71–92.82% across the examined model–event combinations, whereas humanitarian classification ranges from 63.33 to 79.98% and damage-severity assessment from 36.09 to 58.08% (Table 19). No model leads on every task–event combination. The comparatively weak and variable damage-severity results, together with the discrepancy between accuracy and macro-F1 on imbalanced event-specific test sets, limit claims of general robustness. This analysis holds out only three of the seven CrisisMMD events, uses one training seed per condition, and reuses configurations previously selected using mixed-event development data. It therefore provides exploratory evidence of supervised transfer to the specified events, not fully event-blind model selection or demonstrated generalization to independent disaster datasets.

### 5.4. Computational Trade-Offs and Practical Implications

Hardware-matched profiling illustrates that classification performance and inference cost must be considered jointly. On the evaluated NVIDIA A100, CLIP has the lowest warmed-up batch-size-one model-forward latency (34.18–34.25 ms) and inference-process memory use (approximately 2.20 GiB). LLM2CLIP requires 81.70–84.18 ms and approximately 15.73 GiB, while LLaVA-enhanced CLIP requires 1.19–1.31 s and approximately 15.23–15.32 GiB when its image-conditioned generation stage is included without cache reuse (Table 22 and Table 23). LLM2CLIP nevertheless achieves the highest measured throughput with batches of 32. This difference illustrates that batched throughput and single-request latency characterize distinct operating conditions and should not be conflated.

These measurements establish resource requirements for the tested pipelines, not deployment-level performance. Model-forward timings exclude image loading, CPU preprocessing, network transport, queuing, and application orchestration; no concurrent-service or load testing is conducted. The recorded FLOPs/MACs omit unsupported operations and are only partial profiler estimates (Table 24). Moreover, the LLaVA generator is absent from downstream CLIP training but required for uncached inference, so its downstream training-memory measurement does not represent the complete generation-and-classification pipeline. The evidence therefore supports a hardware-specific latency–throughput–memory trade-off rather than claims of computational lightness, real-time deployment, or production scalability.

### 5.5. Limitations and Scope of the Findings

Several aspects of the experimental design constrain causal interpretation and external validity. The statistical comparisons are exploratory rather than confirmatory: the Holm correction jointly applied to all nine within-task model contrasts yields no significant difference. In particular, the paired statistical sensitivity analysis uses only five seed blocks: exact sign-flip tests have limited attainable *p*-value resolution, the paired bootstrap intervals are descriptive and unadjusted for multiplicity, and repeated evaluation of the same TEST records does not estimate variation across independent disaster populations. Although the main CLIP and LLM2CLIP pipelines use 336×336 inputs, their pretrained visual weights, text pathways, and selected adaptation settings differ; their performance differences therefore cannot be attributed solely to LLM-derived text representations. The separate frozen-feature probes control the visual features and downstream classifier capacity but do not reproduce the final partially fine-tuned configurations. The resolution comparison also changes pretrained checkpoints as well as image size. Likewise, the fusion and loss/sampling alternatives were compared primarily on development data with limited seeds rather than through parameter-matched, repeated held-out test experiments.

Damage-severity assessment has additional limitations because Task 3 labels are derived from images and no independently annotated tweet-level severity target is available. In the released partitions, 62 tweet identifiers occur in both TRAIN and TEST despite non-overlapping image IDs; one image–tweet pair also has an identifier inconsistency whose semantic correspondence could not be independently verified. The tweet-disjoint retraining protocol removes measured cross-partition identifier overlap, but it changes TRAIN/DEV membership, reuses the original TEST records, and retains configurations selected under the released protocol. Its results therefore cannot isolate the causal effect of overlap, and the identifier checks do not exclude visually near-duplicate images or semantically paraphrased tweets (Section 4.6). Separately, the matched frozen CLIP image-plus-tweet control produces only a +0.08-point mean macro-F1 change relative to image-only over five seeds (Table 10); no corresponding LLM2CLIP control is completed, and neither frozen condition reproduces the partially fine-tuned benchmark. The nine-pair Affected individuals TEST support similarly limits conclusions about recognition of rare humanitarian categories.

Finally, comparisons with published CrisisMMD studies provide context rather than universally controlled rankings. The original agreed-label benchmark defines a relevant historical reference, but later studies may differ in data partitions, class taxonomies, input modalities, supervision, and metric averaging (Table 25, Table 26 and Table 27). Our three internally evaluated model families share the task-specific records and label definitions; identical experimental conditions have not been established for every external baseline. Taken together, the study offers evidence about the behavior of established pretrained pipelines under the stated CrisisMMD protocols, while leaving questions of mechanism-level causality, generalization beyond the three examined events, and deployment readiness for future investigation.

## 6. Conclusions

This study compared three existing pretrained vision–language pipelines—CLIP, LLM2CLIP, and LLaVA-enhanced CLIP—across informativeness, humanitarian, and image-derived damage-severity classification on CrisisMMD. CLIP was selected by development performance for Tasks 1 and 2, and LLaVA-enhanced CLIP for Task 3. Although LLM2CLIP had the highest observed mean TEST macro-F1 on Tasks 1 and 3, none of the nine model-family contrasts was statistically significant under exact seed-blocked testing with Holm correction (minimum adjusted p=0.5625). These results characterize complete pretrained pipelines under the evaluated protocols; differences in visual checkpoints and adaptation settings prevent attribution to the text representations alone.

The single-seed development comparisons identified concatenation as a competitive fusion choice but did not establish superiority for a fusion or class-imbalance strategy. Controlled frozen-feature probes for Tasks 1–2 likewise showed no consistent predictive advantage for LLM2CLIP-derived text under matched visual-feature conditions. These probes do not reproduce the partially fine-tuned final configurations. Class-level results identified persistent difficulties for the small humanitarian Affected individuals category and the intermediate Mild damage class.

The released damage-severity partitions contain repeated tweet identifiers across training and evaluation. Five-seed tweet-disjoint retraining on the unchanged 529-pair TEST set yielded macro-F1 of 63.80±0.68% for CLIP, 62.46±1.43% for LLM2CLIP, and 63.55±1.32% for LLaVA-enhanced CLIP. Because training/development membership and checkpoint selection conditions differ between protocols, these changes do not isolate the effect of repeated tweets. In a separate matched frozen CLIP control, adding original tweet text changed five-seed macro-F1 only from 60.99±1.25% to 61.07±1.40%; this does not establish the effect of text in the final fine-tuned systems.

Under the shared image–text retrieval protocol, CLIP achieved greater matched–mismatched similarity separation and higher recall than LLM2CLIP. LLaVA rewriting improved retrieval diagnostics within its own image-conditioned pipeline, but the generator observed the paired evaluation image. Exploratory event-held-out experiments showed more variable transfer for humanitarian and severity classification, while hardware profiling revealed higher single-example inference costs for the LLM-based pipelines. These findings motivate matched end-to-end modality ablations, multi-seed evaluation on independent disasters, and realistic deployment-level profiling. The principal contribution is a task-specific empirical assessment of established approaches, not a new architecture or evidence of universal performance superiority.

## Figures and Tables

**Table 1:** Mapping from the original humanitarian annotation categories to the released five-class CrisisMMD Task 2 benchmark. The final experiments use the provided five-class split files without additional category consolidation.

Original Annotation Category	Released Five-Class Label
Affected individuals	Affected individuals
Injured or dead people	Affected individuals
Missing or found people	Affected individuals
Infrastructure and utility damage	Infrastructure and utility damage
Vehicle damage	Infrastructure and utility damage
Rescue, volunteering, or donation effort	Rescue, volunteering, or donation effort
Other relevant information	Other relevant information
Not humanitarian	Not humanitarian

**Table 2:** Image–tweet pair counts by task, class, and released dataset partition. Counts refer to paired image records rather than unique tweet identifiers. Task 3 uses image-derived severity labels with contextual tweet input.

Class	Train	Dev	Test	Total
**Task 1: Informativeness**				
Informative	6345	1056	1030	8431
Not informative	3256	517	504	4277
Task 1 total pairs	9601	1573	1534	12,708
**Task 2: Humanitarian classification**				
Affected individuals	71	9	9	89
Rescue/volunteering/donation	912	149	126	1187
Infrastructure and utility damage	612	80	81	773
Other relevant information	1279	239	235	1753
Not humanitarian	3252	521	504	4277
Task 2 total pairs	6126	998	955	8079
**Task 3: Damage severity (image-derived labels)**				
Little or no damage	333	71	71	475
Mild damage	587	126	126	839
Severe damage	1548	332	332	2212
Task 3 total pairs	2468	529	529	3526

**Table 3:** LLaVA-enhanced CLIP generation and downstream classification settings used in the final experiments. The same image-conditioned prompt and deterministic decoding protocol are applied across tasks.

Setting	Implemented Configuration
Generator checkpoint	llava-hf/llava-1.5-7b-hf (frozen)
Generator processor	Same checkpoint; use_fast = False
Prompt	Exact instruction reproduced above
Generator inputs	Original paired RGB image + normalized tweet
Prompt construction	User role with image and text; processor chat template
Decoding	Greedy; do_sample = False, num_beams = 1
Generation length/batch	64 new tokens/1 image–tweet pair
Generated text	Remove prompt tokens; skip special tokens; collapse whitespace; use normalized original if empty
CLIP classifier input	Original image (336 × 336) + generated text (maximum 77 tokens)
Downstream adaptation	CLIP last 7 visual and text blocks + concatenation/MLP; LLaVA frozen
Recorded runtime	PyTorch 2.9.0+cu126; CUDA 12.6; NVIDIA A100-SXM4-40GB
Transformers requirement	4.44.2 specified; installed run-time version not archived

**Table 4:** Pretrained model configurations and publicly listed checkpoint revisions. The LLaVA revision was the latest publicly available as of October 2026; the exact model and processor revisions used in the original experiments were not recorded. Final benchmark, profiling, and cross-event evaluations used 336×336 visual inputs; 224×224 variants were evaluated on DEV only. Optimization settings are given in Table 6.

Component	CLIP	LLM2CLIP	LLaVA-Enhanced CLIP
Text encoder	OpenAI CLIP text tower	Pretrained LLM2CLIP Llama-3-8B (LLM2Vec) and frozen text adapter	OpenAI CLIP text tower on image-conditioned rewritten tweet
Text tokenizer	Matching CLIP tokenizer	LLM2Vec/tokenizer of pretrained Llama-3-8B backbone	Matching CLIP tokenizer for rewritten tweet
Visual checkpoint (exact HF ID)	openai/clip-vit- large-patch14-336	microsoft/ LLM2CLIP- Openai-L-14-336	openai/clip-vit- large-patch14-336
Visual model revision	ce19dc912ca5cd21c8a653c79e251e808ccabcd1	92512331f393a003c3d98404677f991c188162c9	ce19dc912ca5cd21c8a653c79e251e808ccabcd1
Text model/tokenizer revision	ce19dc912ca5cd21c8a653c79e251e808ccabcd1	1c201fa1d579d0b3ef14b2730651073e9060ab7f	ce19dc912ca5cd21c8a653c79e251e808ccabcd1
Image processor revision	ce19dc912ca5cd21c8a653c79e251e808ccabcd1	ce19dc912ca5cd21c8a653c79e251e808ccabcd1	ce19dc912ca5cd21c8a653c79e251e808ccabcd1
Custom model-code revision	—	e1f6c48ef4f2da0d47b35607e4d46bffaab570c8	—
LLaVA public revision (October 2026)	—	—	b234b804b114d9e37bb655e11cbbb5f5e971b7a9
Downstream visual input	336 × 336	336 × 336	336 × 336 (CLIP stage)
Maximum text length	77	512	77
Additional pretrained model	None	Microsoft pretrained frozen text adapter	Frozen LLaVA-1.5-7B generator
Fine-tuning depth	Last 5 blocks in both CLIP towers	Last 3 blocks in LLM2Vec and visual towers; adapter frozen	Last 7 blocks in downstream CLIP image/text towers; LLaVA frozen
Text input pathway	Cleaned tweet; matching CLIP tokenizer	Cleaned tweet; LLM2Vec mean pooling, then pretrained adapter	Frozen LLaVA image-conditioned rewrite; then CLIP tokenizer
Generative prompt/decoding	None	None	Image-conditioned prompt; greedy decoding (Table 3)
Feature fusion	Concatenation	Concatenation	Concatenation
Classifier	512-unit MLP head	512-unit MLP head	512-unit MLP head (not the LLaVA generator)
Imbalance strategy	Weighted CE	Weighted CE	Balanced sampling + ordinary CE

**Table 5:** Training and evaluation settings for the adaptation and fusion experiments.

Parameter	Value/Notes
Dataset	CrisisMMD agreed-label image–tweet pairs (Tasks 1 and 2); paired image–tweet samples for Task 3
Normalization	L2 normalization per modality
Feature fusion	Concatenation, two-weight learnable fusion, or feature-wise gated fusion; concatenation in final evaluation
Classifier	Linear–512–ReLU–Dropout(0.3)–Linear to class logits
Imbalance variants	Class-weighted cross-entropy, class-weighted focal loss, or class-balanced sampling with cross-entropy
Optimizer	AdamW
Fine-tuning policy	Frozen, full, or final *N* transformer blocks; selected depths depend on pretrained model
Mixed precision	CUDA autocast; FP16 loss scaling when applicable
Selection criterion	Best development macro-F1
Early stopping	Patience = 10
Batch size	32 in the fusion-ablation and final five-seed evaluations
Maximum epochs	100 in the fusion-ablation and final five-seed evaluations
Final evaluation	Five seeds; held-out test scores summarized by mean and sample standard deviation
Metrics	Accuracy, balanced accuracy, macro precision/recall/F1 and additional diagnostic measures

**Table 6:** Selected configurations for the five-seed held-out test evaluation. Every model–task run uses the model-family-specific final 336×336 visual checkpoint in Table 4, concatenation, batch size 32, and MLP dropout 0.3; no 224×224 run contributes to these scores.

Model	Fine-Tuned Blocks	Backbone LR	Classifier LR	Weight Decay	Imbalance Strategy	Max. Text Length
CLIP	Last 5	$5\times 10^{-7}$	$5\times 10^{-4}$	0.01	Weighted CE	77
LLM2CLIP	Last 3	$1\times 10^{-6}$	$2\times 10^{-3}$	0.01	Weighted CE	512
LLaVA-CLIP	Last 7	$5\times 10^{-6}$	$1\times 10^{-4}$	0	Balanced sampling + CE	77

**Table 7:** Development-set fusion configuration comparison (seed 42). Accuracy, macro precision, macro recall, and macro-F1 are percentages. Bold macro-F1 marks the highest observed value within each model–task combination; configurations differ in downstream classifier parameter counts, and bold does not imply architectural or statistical superiority.

Model	Task	Fusion	Accuracy	Macro-P	Macro-R	Macro-F1
CLIP	1	Concat	93.58	92.45	93.14	**92.78**
	1	Weighted	93.52	92.58	92.75	92.67
	1	Gated	93.32	91.99	93.20	92.55
	2	Concat	90.68	90.55	89.59	89.65
	2	Weighted	89.88	90.34	90.13	**90.08**
	2	Gated	90.68	89.31	90.79	90.02
	3	Concat	71.64	63.45	63.98	**63.61**
	3	Weighted	70.32	63.27	62.46	62.38
	3	Gated	70.70	61.98	64.46	62.98
LLM2CLIP	1	Concat	93.20	91.90	92.96	**92.39**
	1	Weighted	93.39	93.21	91.67	92.38
	1	Gated	92.82	91.43	92.63	91.98
	2	Concat	90.28	86.93	90.55	**88.64**
	2	Weighted	89.88	84.16	88.89	86.35
	2	Gated	89.98	86.77	88.33	87.46
	3	Concat	71.27	65.34	62.30	**62.93**
	3	Weighted	71.64	61.90	60.24	60.94
	3	Gated	72.21	62.72	60.75	61.62
LLaVA-CLIP	1	Concat	92.63	91.55	91.79	**91.67**
	1	Weighted	92.18	90.95	91.46	91.20
	1	Gated	92.05	90.89	91.17	91.03
	2	Concat	89.38	84.48	85.78	**84.96**
	2	Weighted	89.08	83.69	86.26	84.69
	2	Gated	88.88	83.57	86.18	84.59
	3	Concat	71.27	62.84	64.97	63.69
	3	Weighted	72.97	65.79	63.70	**64.42**
	3	Gated	72.02	64.85	63.15	63.54

**Table 8:** Development macro-F1 (%) for class-imbalance strategies at seed 42. Bold indicates the highest value for each model and task, without implying statistical significance. The weighted-CE reference results originate from earlier matching development evaluations; alternative strategies are not compared across five test-set seeds.

Model	Imbalance Strategy	Task 1	Task 2	Task 3
CLIP	Weighted CE	**92.78**	89.65	**63.61**
CLIP	Weighted focal	92.42	**89.80**	63.16
CLIP	Balanced sampler + CE	92.75	89.72	62.75
LLM2CLIP	Weighted CE	92.39	**88.64**	**62.93**
LLM2CLIP	Weighted focal	**92.57**	87.43	61.16
LLM2CLIP	Balanced sampler + CE	92.32	87.85	58.57
LLaVA-CLIP	Weighted CE	91.67	84.96	63.69
LLaVA-CLIP	Weighted focal	91.51	84.75	63.75
LLaVA-CLIP	Balanced sampler + CE	**91.93**	**86.18**	**64.17**

**Table 9:** Frozen-representation DEV macro-F1 (%; mean ± sample SD over seeds 42/123/3407) for Tasks 1–2. CLIP and LLM2CLIP text-pathway probes share a frozen CLIP visual encoder and use TRAIN-fitted PCA. Fusion and loss comparisons are development only.

Task	Text Features	Text Only	Concat	Weighted Fusion	Gated Fusion	Unweighted Concat
1	CLIP	83.93±0.27	91.72±0.27	91.10±0.17	91.28±0.17	91.93±0.26
1	LLM2CLIP	83.93±0.62	91.52±0.20	91.29±0.33	91.20±0.20	91.58±0.19
2	CLIP	74.50±0.30	87.15±1.03	84.54±0.43	85.53±0.19	87.12±0.22
2	LLM2CLIP	72.76±0.51	87.68±0.80	84.97±1.03	85.87±1.21	87.60±1.04

**Table 10:** Tweet-disjoint Task 3 frozen-encoder TEST macro-F1 (%; mean ± sample SD). The standalone-modality results use three seeds; the separately trained matched frozen CLIP comparison uses five seeds. Enriched text is conditioned on the paired image and tweet.

Input Condition	Seeds	TEST Macro-F1 (%)
Standalone input conditions		
Image-only	3	60.21±0.86
Original tweet only	3	49.30±3.21
Image-conditioned enriched text only	3	50.39±0.80
Matched frozen CLIP comparison		
Image-only	5	60.99±1.25
Image + original tweet	5	61.07±1.40

**Table 11:** Five-seed held-out TEST performance for all final model–task configurations, reported as mean ± sample standard deviation (percentage points) over five random seeds (42, 123, 2024, 3407, 777). Macro precision, macro recall, and macro-F1 use equal class weighting. The model shown in bold for each task is selected from the development split, independently of the test results. Published baselines are not included here because comparable multi-seed estimates are not reported for them. Task 3 rows use image–tweet inputs and do not measure the added value of text relative to image-only retraining.

Task	Model	Accuracy (%)	Macro-P (%)	Macro-R (%)	Macro-F1 (%)
1	**CLIP**	93.13±0.54	91.83±0.80	92.93±0.29	92.32±0.55
1	LLM2CLIP	93.29±0.30	92.40±0.46	92.41±0.42	92.39±0.33
1	LLaVA-CLIP	92.03±0.29	90.68±0.43	91.51±0.31	91.07±0.30
2	**CLIP**	90.49±0.56	85.78±1.02	89.75±1.96	87.52±1.09
2	LLM2CLIP	88.46±0.58	82.48±0.86	85.09±1.14	83.64±0.69
2	LLaVA-CLIP	89.15±0.22	80.98±0.78	89.31±1.34	84.44±0.57
3	CLIP	70.70±0.97	62.95±1.09	64.61±1.98	63.56±1.22
3	LLM2CLIP	70.96±1.55	64.30±1.97	65.47±3.03	64.68±2.37
3	**LLaVA-CLIP**	70.47±0.47	64.27±1.33	64.40±1.26	64.06±0.98

**Table 12:** Task 1 per-class TEST precision, recall, and F1 for the development-selected CLIP model (mean ± sample SD over five training seeds; %). Supports denote unique test image–tweet pairs per run, not fivefold-expanded sample counts.

Informativeness Class	Support	Precision (%)	Recall (%)	F1 (%)
Not informative	504	87.50±2.00	92.34±1.09	89.84±0.65
Informative	1030	96.15±0.48	93.51±1.24	94.81±0.45

**Table 13:** Task 2 per-class precision, recall, and F1 on the same 955 held-out test pairs (CLIP, mean ± sample standard deviation over five seeds; percentages). Support is the number of test image–tweet pairs in each class, not five times that number.

Class	Support	Precision (%)	Recall (%)	F1 (%)
Affected individuals	9	67.94±3.50	84.44±6.09	75.21±3.69
Infrastructure and utility damage	81	92.48±1.94	93.58±1.03	93.01±0.87
Not humanitarian	504	93.56±1.50	89.92±1.66	91.68±0.44
Other relevant information	235	85.82±2.46	91.57±2.03	88.57±0.94
Rescue, volunteering, or donation effort	126	89.11±1.37	89.21±2.89	89.12±1.06

**Table 14:** Task 3 per-class TEST metrics for the development-selected LLaVA-enhanced CLIP (mean ± sample SD over five seeds; %). Support counts distinct test image–tweet pairs per run, not repeated seeds. Labels are image-derived, and associated tweet text is contextual input.

Damage-Severity Class	Support	Precision (%)	Recall (%)	F1 (%)
Little or no damage	71	63.70±3.26	60.85±5.84	62.07±3.17
Mild damage	126	43.99±1.00	53.33±4.64	48.17±2.48
Severe damage	332	85.11±1.24	79.04±1.27	81.95±0.29

**Table 15:** Task 3 tweet-disjoint sensitivity partitions by image-derived severity class (numbers count image–tweet pairs, not unique tweets). TEST remains exactly the released 529-pair partition; 123 released TRAIN/DEV records are excluded because they share tweet IDs with TEST.

Damage-Severity Class	TRAIN	DEV	TEST	Retained Total
Little or no damage	307	76	71	454
Mild damage	541	136	126	803
Severe damage	1451	363	332	2146
All classes	2299	575	529	3403

**Table 16:** Task 3 TEST performance under the original released and tweet-disjoint evaluation protocols (mean ± sample SD over five seeds; percentages). Both protocols evaluate the same 529 TEST pairs. Δ is the tweet-disjoint minus original mean in percentage points and does not isolate the effect of repeated tweet identifiers.

Model	Metric	Original (%)	Tweet-Disjoint (%)	Δ (pp)
CLIP	Macro-F1	63.56±1.22	63.80±0.68	+0.24
	Accuracy	70.70±0.97	71.08±0.79	+0.38
	Balanced accuracy	64.61±1.98	65.41±0.93	+0.80
LLM2CLIP	Macro-F1	64.68±2.37	62.46±1.43	−2.22
	Accuracy	70.96±1.55	68.85±2.09	−2.11
	Balanced accuracy	65.47±3.03	64.09±1.37	−1.38
LLaVA-enhanced CLIP	Macro-F1	64.06±0.98	63.55±1.32	−0.51
	Accuracy	70.47±0.47	71.34±0.76	+0.87
	Balanced accuracy	64.40±1.26	63.05±1.47	−1.35

**Table 17:** Class-specific TEST F1 under the tweet-disjoint sensitivity protocol (mean ± sample SD across five seeds; percentages). Support is the number of pairs per TEST run, not multiplied by five.

Damage-Severity Class	Support	CLIP	LLM2CLIP	LLaVA-CLIP
Little or no damage	71	58.62±0.96	57.85±1.28	58.40±2.03
Mild damage	126	49.88±1.37	48.84±3.20	48.58±1.95
Severe damage	332	82.91±0.72	80.69±1.91	83.66±0.22

**Table 18:** Seed-blocked TEST macro-F1 comparisons (five matched seeds; differences and 95% paired bootstrap intervals in percentage points). Exact *p* is the two-sided $2^{5}$ sign-flip test on paired seed differences; Holm-adjusted *p* applies jointly to all nine within-task pairwise contrasts (three model pairs per task). The intervals enumerate all $5^{5}$ paired-seed bootstrap resamples and are not multiplicity adjusted. No exact paired comparison reaches p<0.05; no Holm-adjusted comparison is significant.

Task	Model A − Model B	ΔF1 (pp)	Paired 95% CI (pp)	Exact *p*	Holm-Adjusted *p*
1	CLIP − LLM2CLIP	−0.07	[−0.69,+0.56]	0.8750	1.0000
1	CLIP − LLaVA-CLIP	+1.26	[+0.88,+1.72]	0.0625	0.5625
1	LLM2CLIP − LLaVA-CLIP	+1.33	[+0.95,+1.75]	0.0625	0.5625
2	CLIP − LLM2CLIP	+3.88	[+3.16,+4.50]	0.0625	0.5625
2	CLIP − LLaVA-CLIP	+3.08	[+2.09,+4.06]	0.0625	0.5625
2	LLM2CLIP − LLaVA-CLIP	−0.80	[−1.75,−0.22]	0.0625	0.5625
3	LLaVA-CLIP − CLIP	+0.50	[−1.03,+1.67]	0.5625	1.0000
3	LLaVA-CLIP − LLM2CLIP	−0.62	[−2.93,+1.69]	0.6875	1.0000
3	LLM2CLIP − CLIP	+1.12	[−0.40,+2.67]	0.2500	1.0000

**Table 19:** Unseen-event TEST performance (percent), for seed 42 using previously selected model configurations. *n* counts image–tweet pairs for each held-out disaster and task. Macro-F1 is averaged equally across classes. Bold marks the highest observed macro-F1 among the three models within the same task and held-out event; it does not indicate statistical significance. This event-held-out evaluation uses different training and test partitions from the five-seed official benchmark experiment in Table 11.

			CLIP		LLM2CLIP		LLaVA-CLIP	
Task	Held-Out Event	Test n	Acc.	Macro-F1	Acc.	Macro-F1	Acc.	Macro-F1
1	Hurricane Harvey	3157	94.24	**92.82**	94.11	92.60	92.94	91.43
1	Mexico Earthquake	1114	89.95	**87.25**	87.25	84.95	87.43	84.71
1	California Wildfires	1195	89.62	85.72	91.46	88.14	92.30	**88.94**
2	Hurricane Harvey	1954	89.41	**79.98**	88.13	73.77	88.18	72.24
2	Mexico Earthquake	585	78.97	**73.74**	77.61	73.21	74.70	69.60
2	California Wildfires	511	80.63	67.73	76.71	63.33	80.63	**70.32**
3	Hurricane Harvey	892	66.14	54.40	62.44	52.74	69.17	**58.08**
3	Mexico Earthquake	178	80.34	36.49	85.39	**57.01**	78.65	42.62
3	California Wildfires	531	80.23	43.45	68.74	36.09	83.62	**48.63**

**Table 20:** Exploratory held-out TEST image–text alignment and image-to-text retrieval (seed 42; shared-protocol CLIP–LLM2CLIP comparison and separate LLaVA analysis). Similarities are raw cosine values and recalls are percentages. The alignment gap is matched minus randomly mismatched mean cosine (Equation (13)); larger gaps and recalls indicate stronger separation or retrieval in the evaluated embedding space. LLaVA-CLIP uses image-conditioned enriched text generated from the same image used in the subsequent similarity and retrieval evaluation; its values therefore characterize this image-conditioned pipeline, not independent image–text alignment. The two alignment analyses use different selected model configurations, so this is a descriptive, not hyperparameter-matched, three-family comparison; higher absolute cosine across model families is not itself evidence of superior semantic alignment.

Task	Model	Matched	Mismatched	Gap ↑	R@1 (%)	R@5 (%)	R@10 (%)
1	CLIP	0.2517	0.1004	0.1513	35.59	52.02	59.06
1	LLM2CLIP	0.2833	0.1479	0.1354	24.58	39.90	46.74
1	LLaVA-CLIP (enriched)	0.2463	0.0926	0.1537	38.79	56.84	62.71
2	CLIP	0.2424	0.0562	0.1862	47.75	68.27	74.97
2	LLM2CLIP	0.2840	0.1310	0.1530	37.91	58.43	66.28
2	LLaVA-CLIP (enriched)	0.2271	0.0527	0.1743	48.59	69.84	78.12
3	CLIP	0.2379	0.1124	0.1255	20.98	39.32	52.36
3	LLM2CLIP	0.2829	0.1841	0.0988	14.74	27.41	37.43
3	LLaVA-CLIP (enriched)	0.2126	0.0938	0.1188	22.31	41.02	51.80

**Table 21:** Effect of LLaVA image-conditioned text enrichment on held-out TEST alignment, measured with the same trained downstream CLIP encoders (seed 42). Changes are enriched minus original; retrieval changes are percentage points (pp). Because the rewriting model receives the very image subsequently used for similarity evaluation, positive changes are partly expected by construction; values are descriptive and have not been subjected to multi-seed or paired-sample significance testing.

Task	Gap Original	Gap Enriched	Δ Gap	Δ R@1 (pp)	Δ R@5 (pp)	Δ R@10 (pp)
1	0.1458	0.1537	+0.0080	+6.06	+9.13	+8.47
2	0.1686	0.1743	+0.0058	+6.70	+8.38	+8.06
3	0.1086	0.1188	+0.0102	+5.86	+11.53	+10.78

**Table 22:** Model complexity for the final selected architectures (representative Task 1 counts; Tasks 2–3 differ only slightly in output-head dimensions). Model + buffers is the in-memory parameter/buffer footprint at the measured precision, not a compressed checkpoint-file size. LLaVA-CLIP counts its frozen auxiliary 7B generator, which is required for uncached inference, whereas only the downstream CLIP towers and classification head are optimized.

Model	Total (M)	Trainable (M)	Model + Buffers (GiB)
CLIP	428.73	99.21	1.60
LLM2CLIP	8084.82	693.44	15.06
LLaVA-CLIP	7492.16	138.58	13.96

**Table 23:** Training and uncached TEST inference cost on one NVIDIA A100-SXM4-40GB (seed 42, 336×336 inputs). Epoch time is mean training-only time; total time is cumulative TRAIN + DEV wall time over the epochs actually run, not the end-to-end cost of dataset preparation or frozen LLaVA caption generation. Training peak is PyTorch allocated memory during optimization; inference peak is sampled NVML process memory in a separate optimizer-free process (BS = 1). The inference latency is warmed-up model-forward time (20 runs, BS = 1), and throughput is measured over the complete test set with BS = 32, including uncached image-conditioned generation for LLaVA-CLIP. Neither inference figure includes input image I/O or CPU preprocessing.

Task	Model	Epoch (s)	TRAIN + DEV (min)	Train Peak (GiB)	BS = 1 Latency (ms)	Infer Peak (GiB)	BS = 32 Throughput (Samples/s)
1	CLIP	62.5	20.1	7.35	34.19	2.20	42.50
1	LLM2CLIP	215.2	44.6	22.69	81.70	15.73	142.74
1	LLaVA-CLIP	156.9	67.4	7.92	1243.94	15.24	0.91
2	CLIP	42.6	16.8	7.35	34.18	2.20	42.51
2	LLM2CLIP	102.7	37.7	22.69	84.18	15.73	140.99
2	LLaVA-CLIP	42.1	10.6	7.92	1314.39	15.32	0.92
3	CLIP	45.9	18.4	7.35	34.25	2.20	42.44
3	LLM2CLIP	33.6	8.6	21.73	83.83	15.73	145.53
3	LLaVA-CLIP	22.7	9.1	7.92	1189.68	15.23	0.86

**Table 24:** Partial operation counts reported by PyTorch profiling (supported operations only). FLOPs/MACs are not complete or independently validated totals for the architectures; some fused/custom operations are unsupported and the LLaVA generation workload depends on generated token count. The MAC column is derived as FLOPs/2 under the conventional two-operations-per-multiply-accumulate assumption, not independently measured. Values are indicative, not a hardware-independent basis for ranking models.

Task	Model	Partial TFLOPs	Partial TMACs
1	CLIP	0.36	0.18
1	LLM2CLIP	0.98	0.49
1	LLaVA-CLIP	10.81	5.41
2	CLIP	0.36	0.18
2	LLM2CLIP	0.73	0.36
2	LLaVA-CLIP	10.40	5.20
3	CLIP	0.36	0.18
3	LLM2CLIP	0.84	0.42
3	LLaVA-CLIP	10.33	5.17

**Table 25:** Published CrisisMMD protocol audit. Released denotes explicit use of Ofli et al.’s agreed-label multimodal partitions or matching published counts; it does not mean third-party record IDs were independently audited. Different indicates a documented partition or target mismatch; not established means the primary source does not specify enough detail. T1/T2/T3 denote informativeness/humanitarian/damage-severity classification.

Method (Cited Study)	Task	Split Versus Ours	Labels versus Ours	Learning Regime	Qualification
CNN, VGG16, VGG16 + CNN [18]	1, 2	Released benchmark source	Same released agreed-label targets	Supervised	Defines the T1/T2 multimodal reference partitions.
VGG16 + FastText [21]	1, 3	**Different**: event-stratified splits	T1 binary; T3 image-derived severity	Supervised	Uses study-specific sampling; results moved to Table 26.
RoBERTa + DenseNet [20]	1, 2	Not established	Nominal T1; T2 five-class identity unverified	Supervised	Model-specific numerical attribution insufficient for a comparable benchmark score.
BERT + RegNetY320 [19]	1, 2	**Released**: matching T1/T2 counts	Same five T2 categories	Supervised	Reports 9601/1573/1534 (T1) and 6126/998/955 (T2).
CBAN-Dot [29]	1, 2	Not established	T1/T2 mapping not established	Not established	Original publication does not establish matching partitions.
FixMatchLS [11]	1, 2	**Released**: Ofli split for cited scores	Same five T2 categories	Semi-supervised	Its additional self-defined split experiment is distinct; Table 4 uses released evaluation partitions and extra unlabeled data.
DenseNet121 + BERT [9]	1	Not established	Nominal T1 binary	Supervised	Exact sample and evaluation-partition identity unverified.
DMCC [30]	1, 2	Agreed-label subset stated; exact split not established	T1 binary; **T2 eight classes, not five**	Supervised	Published eight-category T2 differs; released code also references the all-label files. T2 score is contextual (Table 26).
RoBERTaMFT [24]	1–3	Not established	T1/T2/T3 exact mapping unverified	Co-learning; type unverified	Reported multimodal T3 input, but exact samples and training regime unverified.
BERT-LSTM + ResNet50 [8]	1	**Released**: matching counts	Same T1 binary labels	Supervised	Reports the 9601/1573/1534 agreed-label split; published F1 is weighted.
CMB-CLIP-cc [27]	1, 2	Not established	Nominal T1; T2 five-class identity unverified	Supervised	Verified CMB-CLIP-cc macro-F1 values: 90.81% (T1), 78.10% (T2); weighted-F1 and exact split equivalence are separate issues.
LLaVA + RoBERTa [28]	1	**Released**: matching counts	Same T1 binary labels	Supervised	Reports 9601/1573/1534 from the original released evaluation.
Embed + features (text-only) [17]	1	Not established	Nominal T1 binary	Supervised	Input modality differs; split identity unverified.
ViT-16 + GPT-2 + RF [22]	1	**Different**: 13,608/2237/2237	T1 binary; sample population differs	Supervised	Uses the larger all-label Task 1 partitions, not our agreed-label subset; Table 26.
Multihead APM [12]	1, 2	**Released**: Ofli official split	Same five T2 categories	Semi-supervised	Explicitly adopts released multimodal partitions; incorporates 122K unlabeled pairs.
SSE-Cross-BERT-DenseNet (Setting A) [23]	1–3	**Different**: study-specific counts	T1/T2 label agreement filter; T3 image-derived; class mapping not verified equivalent	Supervised + stochastic graph augmentation	Setting A: T1 7876/553/2821; T2 1352/540/1467; T3 2590/340/358 (train/dev/test); outside our numerical matched table.
CLIP-BCA-Gated [26]	2	**Different**: stratified 70/15/15	Study-specific five-category consolidation; identical record IDs not established	Supervised	Reports 91.77% accuracy and 91.74% F1 under alternative split; averaging convention not assumed to be macro.
Text-only BERT + SMOTE [6]	2	**Different**: 70:30	T2 five-class identity unverified	Supervised + SMOTE	Custom split; not a matched Task 2 comparator.
XceptionNet (image-only) [7]	1	**Different**: 80:20	Nominal T1 binary	Supervised	Alternative train/test partition; not a matched Task 1 comparator.
VGG16 + FastText Task 2 [21]	2	**Different**: custom, two-stage	**Modified**: two-stage task	Supervised	Distinct from the five-class humanitarian benchmark.
CLIP, LLM2CLIP, LLaVA-CLIP (this work)	1–3	**Same**: locally verified	**Same**: verified task labels	Supervised	Identical within-study records and task targets; T3 released split has repeated tweet IDs across partitions.

**Table 26:** Published CrisisMMD results under alternative split or label protocols. Abavisani et al. report their Setting A macro-F1; the remaining F1 values retain the original papers’ reporting conventions, which need not equal macro-F1. The results are contextual and are not matched comparisons with our fixed-split TEST scores. A dash denotes an unreported metric.

Published Method/Setting	Task	Accuracy (%)	F1 (%)	F1 Definition
SSE-Cross-BERT-DenseNet (Setting A) [23]	1	89.33	88.09	Macro
	2	91.14	68.41	Macro
	3	72.65	59.76	Macro
VGG16 + FastText (event-stratified split) [21]	1	87.60	87.50	As reported
	3	68.90	67.00	As reported
DMCC (eight-category humanitarian task) [30]	2	88.00	–	Not reported
ViT-16 + GPT-2 + RF (all-label split) [22]	1	84.66	–	Not reported
CLIP-BCA-Gated [26]	2	91.77	91.74	As reported

**Table 27:** Published CrisisMMD results and our five-seed TEST means. Published studies are grouped by whether their evaluation is documented as using the released agreed-label partitions; unresolved cases remain descriptive literature context. External F1 values retain their original reporting conventions, whereas our F1 values are macro-averaged. Record-level identity and evaluation-metric equivalence were not independently verified for external implementations (Table 25); documented alternative-protocol scores appear in Table 26. Our five-seed variability is reported in Table 11. Bold identifies the model selected using DEV macro-F1 for each task, not statistically significant TEST superiority. Task 3 uses the released image-level split, which contains cross-partition tweet overlap (Section 3.1).

Models	Task1				Task2				Task3			
	**Accuracy**	**F1** **(Reported)**	**Precision**	**Recall**	**Accuracy**	**F1** **(Reported)**	**Precision**	**Recall**	**Accuracy**	**F1** **(Reported)**	**Precision**	**Recall**
Published results using the released agreed-label Tasks 1–2 evaluation protocol												
CNN (Text only) [18]	80.80%	80.90%	81.00%	81.00%	70.40%	67.70%	70.00%	70.00%	-	-	-	-
VGG-16 (Image only) [18]	83.30%	83.20%	83.10%	83.30%	76.80%	76.30%	76.40%	76.80%	-	-	-	-
VGG-16 + CNN [18]	84.40%	84.20%	84.10%	84.00%	78.40%	78.30%	78.50%	78.00%	-	-	-	-
BERT + RegNetY320 [19]	89.63%	89.63%	89.63%	89.63%	86.59%	86.59%	86.59%	86.59%	-	-	-	-
FixMatchLS [11]	-	90.50%	91.00%	90.80%	-	88.10%	88.50%	88.10%	-	-	-	-
BERT-LSTM + ResNet50 [8]	91.00%	91.00%	93.00%	94.00%	-	-	-	-	-	-	-	-
LLaVA + RoBERTa Text & Caption [28]	91.40%	90.20%	-	-	-	-	-	-	-	-	-	-
Multihead APM Agree No Low [12]	91.23%	91.22%	91.21%	91.27%	89.55%	89.55%	89.73%	89.58%	-	-	-	-
Published results for which exact split equivalence remains unresolved												
CBAN-Dot [29]	92.63%	92.67%	-	-	88.38%	88.40%	-	-	-	-	-	-
DenseNet121 + BERT [9]	85.33%	85.09%	-	-	-	-	-	-	-	-	-	-
DMCC [30]	92.24%	-	92.23%	92.24%	-	-	-	-	-	-	-	-
RoBERTaMFT w/Score Fusion [24]	88.90%	87.40%	-	-	84.90%	80.40%	-	-	70.30%	58.70%	-	-
CMB-CLIP-cc [27]	92.03%	90.81%	-	-	88.27%	78.10%	-	-	-	-	-	-
Embed + features (Text-Only) [17]	87.93%	87.62%	87.70%	87.93%	-	-	-	-	-	-	-	-
This study: five-seed test means (development-selected models indicated by bold macro-F1)												
CLIP (ours)	93.13%	**92.32%**	91.83%	92.93%	90.49%	**87.52%**	85.78%	89.75%	70.70%	63.56%	62.95%	64.61%
LLaVA-CLIP (ours)	92.03%	91.07%	90.68%	91.51%	89.15%	84.44%	80.98%	89.31%	70.47%	**64.06%**	64.27%	64.40%
LLM2CLIP (ours)	93.29%	92.39%	92.40%	92.41%	88.46%	83.64%	82.48%	85.09%	70.96%	64.68%	64.30%	65.47%

## Data Availability

The data used in this study are publicly available through the CrisisMMD dataset release cited in the manuscript. Additional implementation details are available from the corresponding author upon reasonable request.

## References

[1] Ritchie H., Rosado P., Roser M. (2022). Natural Disasters. Our World in Data. https://ourworldindata.org/natural-disasters.

[2] Chiu Y.Y., Omura H., Chen H.E., Chen S.C. (2020). Indicators for Post-Disaster Search and Rescue Efficiency Developed Using Progressive Death Tolls. Sustainability.

[3] World Health Organization (2025). WHO Health Emergency Appeal, 2025.

[4] Hunt K., Agarwal P., Zhuang J. (2022). Monitoring misinformation on Twitter during crisis events: A machine learning approach. Risk Anal..

[5] Hunt K., Wang B., Zhuang J. (2020). Misinformation debunking and cross-platform information sharing through Twitter during Hurricanes Harvey and Irma: A case study on shelters and ID checks. Nat. Hazards.

[6] Jain T., Gopalani D., Meena Y.K. Classification of Humanitarian Crisis Response Through Unimodal Multi-Class Textual Classification. Proceedings of the 2024 International Conference on Emerging Systems and Intelligent Computing (ESIC).

[7] Jain T., Gopalani D., Meena Y.K. Image Tweet Classification for Crisis Informative Task. Proceedings of the 2024 International Conference on Integrated Circuits, Communication, and Computing Systems (ICIC3S).

[8] Tasneem F., Chakraborty S., Chy A.N. An Early Synthesis of Deep Neural Networks to Identify Multimodal Informative Disaster Tweets. Proceedings of the 20th International Conference on Information Systems for Crisis Response and Management (ISCRAM 2023).

[9] Kamoji S., Kalla M., Joshi C. Fusion of Multimodal Textual and Visual Descriptors for Analyzing Disaster Response. Proceedings of the 2023 5th International Conference on Smart Systems and Inventive Technology (ICSSIT).

[10] Madichetty S., M S. (2020). Classifying informative and non-informative tweets from the twitter by adapting image features during disaster. Multimed. Tools Appl..

[11] Sirbu I., Sosea T., Caragea C., Caragea D., Rebedea T. Multimodal Semi-supervised Learning for Disaster Tweet Classification. Proceedings of the 29th International Conference on Computational Linguistics.

[12] Sîrbu I., Popovici R.A., Rebedea T., Trăuşan-Matu Ş. (2025). Multihead Average Pseudo-Margin Learning for Disaster Tweet Classification. Information.

[13] Radford A., Kim J.W., Hallacy C., Ramesh A., Goh G., Agarwal S., Sastry G., Askell A., Mishkin P., Clark J. (2021). Learning Transferable Visual Models From Natural Language Supervision. arXiv.

[14] Huang W., Wu A., Yang Y., Luo X., Yang Y., Hu L., Dai Q., Dai X., Chen D., Luo C. (2024). LLM2CLIP: Powerful Language Model Unlock Richer Visual Representation. arXiv.

[15] Liu H., Li C., Wu Q., Lee Y.J. (2023). Visual Instruction Tuning. arXiv.

[16] Alam F., Ofli F., Imran M. (2018). CrisisMMD: Multimodal Twitter Datasets from Natural Disasters. arXiv.

[17] Jain T., Gopalani D., Meena Y.K. (2025). Informative task classification with concatenated embeddings using deep learning on crisisMMD. Int. J. Comput. Appl..

[18] Ofli F., Alam F., Imran M. (2020). Analysis of Social Media Data using Multimodal Deep Learning for Disaster Response. Proceedings of the 17th International Conference on Information Systems for Crisis Response and Management (ISCRAM 2020), Blacksburg, VA, USA.

[19] Kota S., Haridasan S., Rattani A., Bowen A., Rimmington G., Dutta A. Multimodal Combination of Text and Image Tweets for Disaster Response Assessment. Proceedings of the International Workshop on Data-Driven Resilience Research 2022 (D2R2), CEUR-WS.org.

[20] Adwaith D., Abishake A.K., Raghul S.V., Sivasankar E. (2022). Enhancing multimodal disaster tweet classification using state-of-the-art deep learning networks. Multimed. Tools Appl..

[21] Zou Z., Gan H., Huang Q., Cai T., Cao K. (2021). Disaster Image Classification by Fusing Multimodal Social Media Data. ISPRS Int. J. Geo-Inf..

[22] Gite S., Patil S., Pradhan B., Yadav M., Basak S., Rajendra A., Alamri A., Raykar K., Kotecha K. (2025). Analysis of Multimodal Social Media Data Utilizing VIT Base 16 and GPT-2 for Disaster Response. Arab. J. Sci. Eng..

[23] Abavisani M., Wu L., Hu S., Tetreault J., Jaimes A. Multimodal Categorization of Crisis Events in Social Media. Proceedings of the IEEE/CVF Conference on Computer Vision and Pattern Recognition (CVPR).

[24] Yoon J., Choi G., Choi C. (2023). Multimedia analysis of robustly optimized multimodal transformer based on vision and language co-learning. Inf. Fusion.

[25] Dinani S.T., Caragea D. Disaster Image Classification Using Pre-trained Transformer and Contrastive Learning Models. Proceedings of the 2023 IEEE 10th International Conference on Data Science and Advanced Analytics (DSAA).

[26] Li S., Liu Q., Pan Z., Wu X. (2025). CLIP-BCA-Gated: A Dynamic Multimodal Framework for Real-Time Humanitarian Crisis Classification with Bi-Cross-Attention and Adaptive Gating. Appl. Sci..

[27] Farah B., Cleuziou G., Gracianne C., Hafiane A., Halftermeyer A., Canals R. (2024). Image-text crisis tweet categorization: A caption-based approach. Proc. Int. ISCRAM Conf..

[28] Giaccaglia P., Bono C.A., Pernici B. Enhancing Emergency Post Classification through Image Information Amplification via Large Language Models. Proceedings of the Information Systems for Crisis Response and Management (ISCRAM 2024).

[29] Cheung T.-h., Lam K.-m. (2022). Crossmodal bipolar attention for multimodal classification on social media. Neurocomputing.

[30] Rezk M., Elmadany N., Hamad R.K., Badran E.F. (2023). Categorizing Crises From Social Media Feeds via Multimodal Channel Attention. IEEE Access.

